# Gambling in Young Adults Aged 17–24 Years: A Population-Based Study

**DOI:** 10.1007/s10899-020-09948-z

**Published:** 2020-04-18

**Authors:** Linda Hollén, Rita Dörner, Mark D. Griffiths, Alan Emond

**Affiliations:** 1 Centre for Academic Child Health, Bristol Medical School, Bristol, BS8 1NU UK; 2 Institute of Tropical Medicine and International Health, Charité Universitätsmedizin Berlin, Berlin, Germany; 3 International Gaming Research Unit, Psychology Department, Nottingham Trent University, Nottingham, UK

**Keywords:** Adolescent gambling, Youth gambling, Gambling antecedents, Internet gambling, Longitudinal study, ALSPAC

## Abstract

A large contemporary UK cohort study, the Avon Longitudinal Study of Parents and Children, was used to investigate gambling behavior and to explore the antecedents of regular gambling in the 17–24-year age group. Participants completed computer-administered gambling surveys in research clinics, on paper, and online. The sample sizes were 3566 at age 17 years, 3940 at 20 years, and 3841 at 24 years; only 1672 completed all three surveys. Participation in gambling in the last year was reported by 54% of 17-year-olds, rising to 68% at 20 years, and 66% at 24 years, with little overall variance. Regular (weekly) gambling showed a strong gender effect, increasing among young men from 13% at 17 years to 18% at 20 years, and 17% at 24 years. Although gambling frequency increased between the ages of 17 and 20 years, gambling behaviors showed little variance between 20 and 24 years, except online gambling and betting on horseraces. The commonest forms of gambling were playing scratchcards, playing the lottery, and private betting with friends. Gambling on activities via the internet increased markedly between 17 and 24 years, especially among males. In the fully adjusted model, individual antecedents of regular gambling were being male, and having a low IQ, an external locus of control, and high sensation seeking scores. Parental gambling behavior and maternal educational background were associated with regular gambling in both sexes. Regular gambling was associated with smoking cigarettes and frequent and harmful use of alcohol, but no associations with depression were found.

**Electronic supplementary material:**

The online version of this article (10.1007/s10899-020-09948-z) contains supplementary material, which is available to authorized users.

## Introduction

In the Health Survey for England in 2018 (NHS Digital 2019), 57% of adult men and 54% of adult women reported gambling in the past year. In the age range 16–24 years, 45% of men and 33% of women reported gambling in the past year, and 20% of men and 2% of women gambled online. The increased availability of gambling and the expansion of opportunities to gamble online have led to increases in the number of young people who gamble on a regular basis (Calado et al. 2017; Griffiths and Parke 2010). Studies from other industrialized countries (Molinaro et al. 2014) indicate that in spite of adolescent gambling being an illegal activity, in many countries youth engage in gambling with a prevalence rate higher than adults (Calado et al. 2017; Volberg et al. 2010). Contemporary youth have grown up in an era where opportunities to gamble are increasingly widespread (Volberg et al. 2010), and remote forms of gambling via smartphone and the internet are making it easier to access gambling than previous generations (Griffiths and Parke 2010).

Young people are known to be at risk of problems with gambling because of cognitive immaturities, such as illusions of control over outcomes (Chambers and Potenza 2003), and poor understanding of statistical probability (Delfabbro et al. 2006). These can lead to chasing losses, a common gambling problem. In adolescence, executive function is not fully developed, which increases impulsivity and risk-taking behaviors (Blakemore and Choudhury 2006). This immaturity in self-regulation can increase the frequency of placing bets impulsively, especially in-game sports betting. Young people may also have heightened susceptibility to environmental factors that can determine gambling, including family and peer influences (Langhinrichsen-Rohling et al. 2004), and messages from marketing campaigns that distort the social and financial rewards from gambling (Derevensky et al. 2010). Sports betting is widely advertised, especially aimed at young men (Lopez-Gonzalez et al 2017). Gambling is now being promoted to children and adolescents via social media. A recent study of gambling marketing on *Twitter* (Smith et al. 2019) demonstrated how adolescents are being exposed to gambling advertising and are sharing and retweeting messages from gambling companies.

Most studies of youth gambling are cross-sectional, and few have examined gambling patterns among adolescents and long-term implications in adulthood. Longitudinal studies which have followed adolescents across the transition to adulthood include investigations in Canada (Vitaro et al. 2001), Australia (Delfabbro et al. 2014), and the U.S. (Bray et al. 2014; Slutske et al. 2005; Winters et al. 2002). Some of these studies (e.g., Delfabbro et al. 2014; Winters et al. 2002) have addressed specific questions relating to stability or change in gambling and problem gambling across adolescence and early adulthood. These studies have suggested that rates of gambling increase gradually with age, and particularly in the transition from adolescence to young adulthood. In this age group, gambling is increasing for some activities (e.g., online betting) while decreasing for others (e.g., card games). Although prior gambling is predictive of subsequent behavior, there is considerable within-person inconsistency, and preferences for different types of games are highly variable from one year to the next (Delfabbro et al. 2014).

Young people’s gambling behavior reflects a complicated interaction between genetic risk, demographic factors, family gambling habits, and developmental traits such as impulsivity. To investigate these interactions over time, a large longitudinal study is required, with demographic, environmental and genetic information on the participants. The Avon Longitudinal Study of Parents and Children (ALSPAC) is a large contemporary British cohort study which fulfils these criteria and provides a unique opportunity to investigate gambling in late adolescence and early adult life. This paper summarizes young people’s gambling activity at 17 years, 20 years, and 24 years, and investigates the individual, familial, and environmental antecedents of regular gambling during this critical developmental period. It was hypothesized that (1) gambling frequency would increase between 17 and 24 years because of increased availability of legal gambling opportunities and increased income, and (2) regular gambling at these ages would be associated with both family factors (such as history of parental gambling) and individual developmental traits (such as hyperactivity and sensation seeking).

## Methods

### ALSPAC Cohort

The Avon Longitudinal Study of Parents and Children (ALSPAC) is a birth cohort study which enrolled mothers in early pregnancy in the Bristol and surrounding areas in England in 1991–1992 (www.bris.ac.uk/alspac). The mothers enrolled in the study were representative of the population of pregnant women in the UK at that time. ALSPAC has detailed information on parents (Fraser at al 2012) and children (Boyd et al 2013), collected prospectively at multiple times during pregnancy and throughout childhood. Sources of data include self-report surveys, clinical assessments, birth, medical, and educational records, and biological samples. The initial recruitment resulted in a core cohort of 14,541 pregnancies and 13,988 children alive at 12 months.

A total of 913 additional participants have been enrolled in the study since the age of 7 years with 195 of these joining since the age of 18 years. This additional enrolment provides a baseline sample of 14,901 participants who were alive at one year of age. The study website contains details of all the data that are available through a fully searchable data dictionary (https://www.bris.ac.uk/alspac/researchers/data-access/data-dictionary/). Ethical approval for the ALSPAC was obtained from local research ethics committees. Informed consent for the use of data collected via questionnaires and clinics was obtained from participants following the recommendations of the ALSPAC Ethics and Law Committee at the time.

### Gambling Participants

Participants in the ALSPAC were invited to attend a research clinic in Bristol (south-west England) when they were approximately 17-years old (2009–2011). At the clinic, participants completed a confidential computer-administered survey about their gambling experiences, and their attitudes to gambling and risk-taking. Young people who were invited but did not attend the clinic were encouraged to complete the same survey available online on the University of Bristol website. Two more surveys were sent out as part of ALSPAC when the young people were aged 20 years (2012–2013) and 24 years (2016–2017). Although the gambling sections followed a similar format to the one at 17 years, these were part of a bigger survey with five to ten sections on other aspects of the participants’ life. At the latter two ages, the survey was available to complete online or in paper format. If a respondent completed a paper survey, the data were entered into the online REDCap database by ALSPAC staff.

### Gambling Activity

Participation in gambling during the past year was assessed at all three time points using items derived from the British Gambling Prevalence Survey 2007 (Wardle et al. 2007). At age 17 and 24 years, 17 items were used, and at age 20 years, 16 items. For the purpose of the analyses, these items were reduced to 13 for all time points (Table 1). The response options at all ages were collapsed to 0 (“no gambling within the past 12 months”), 1 (“less than weekly gambling within the past 12 months), and 2 (“weekly gambling or more within the past 12 months”) and used as outcomes in subsequent analyses. Only participants who answered all 13 questions in the analyses were used (97%). Those who answered *no gambling within the past 12 months* on all 13 questions were classified as non-gamblers. Young people gambling less than weekly on at least one of the 13 activities are hereafter referred to as *occasional gamblers* and those gambling weekly or more frequently on at least one of the 13 activities are referred to as *regular gamblers*.

**Table 1:** Gambling activities included in the analyses at the three time points

Activity	Include	Exclude
1. Lottery games	*Lotto, Thunderball* and *Euromillions*	Scratchcards
2. Scratchcards	Lottery scratchcard games played offline and online	Newspaper or magazine scratchcards
3. Football pools	–	Betting on football matches with a bookmaker
4. Bingo cards or tickets	Playing boards at a bingo hall	Newspaper bingo tickets, or bingo played online
5. Fruit (slot) machines	–	Quiz machines
6. Virtual gaming machines	Betting on virtual roulette, keno, bingo etc. in a bookmaker’s	Quiz machines
7. Table games	Roulette, dice, poker, or cards in a casino	Poker or casino games played online
8. Online gambling	Playing poker, bingo, slot machine style games, or casino games for money online through a computer, mobile phone or interactive television	Bets made with online bookmakers or betting exchanges
9. Online betting with a bookmaker	Betting online through a computer, mobile phone or interactive TV on any event or sport	Bets made with a betting exchange or spread-betting
10. Betting exchange	Peer to peer betting	–
11. Betting on horse races	Betting on horse races with a bookmaker, by phone, or at the track. Also includes tote betting and betting on virtual horse races shown in a bookmaker’s	Bets made with online bookmakers or betting exchanges
12. Spread-betting		–
13. Private betting	Playing cards or games for money with friends, family or colleagues	–

### Associations of Gambling

The choice of variables used was informed by previous analyses and the gambling literature, and were clustered into child, parental, and socio-economic factors. Variables used were limited to those previously collected at different ages in ALSPAC. Child variables included: gender, IQ (collected at age 8 years), playing videogames (at age 13 years), hyperactivity and conduct problems (at age 16.5 years), locus of control (at age 16.5 years), sensation seeking (at age 17 years), stressful life events (at age 16 years), education/employment status (at age 17 and 20 years), diagnosed depression (at age 17 years), smoking and alcohol use (at age 16.5, 21 and 23 years), and social media use (at age 24 years).

Parental variables used were: maternal age at birth, maternal highest education level (collected in pregnancy), maternal gambling (recorded when child was aged six and 18 years), paternal gambling (when child was aged 6 years), and maternal and paternal depression (when child was aged 10–12 years). Household/socioeconomic status (hereafter SES) variables used were: crowding index in pregnancy, financial difficulties in pregnancy, index of multiple deprivation when child was aged 11 years, and housing status when child was aged 18 years. A detailed description of all variables and instruments used can be found in supplementary Methods 1.

### Statistical Analyses

The statistical analyses underwent several stages. Due to the longitudinal nature of the data, the research team first tried to implement trajectory analyses using various methods in Mplus v.8.1 (Múthen and Múthen 2019), including latent class analysis with the categorical gambling activities. The outcomes of all these attempts were models with very poor statistical fit and there was very minor slope heterogeneity over time, making these models of little use (more details available upon request).

**Fig. 1:**
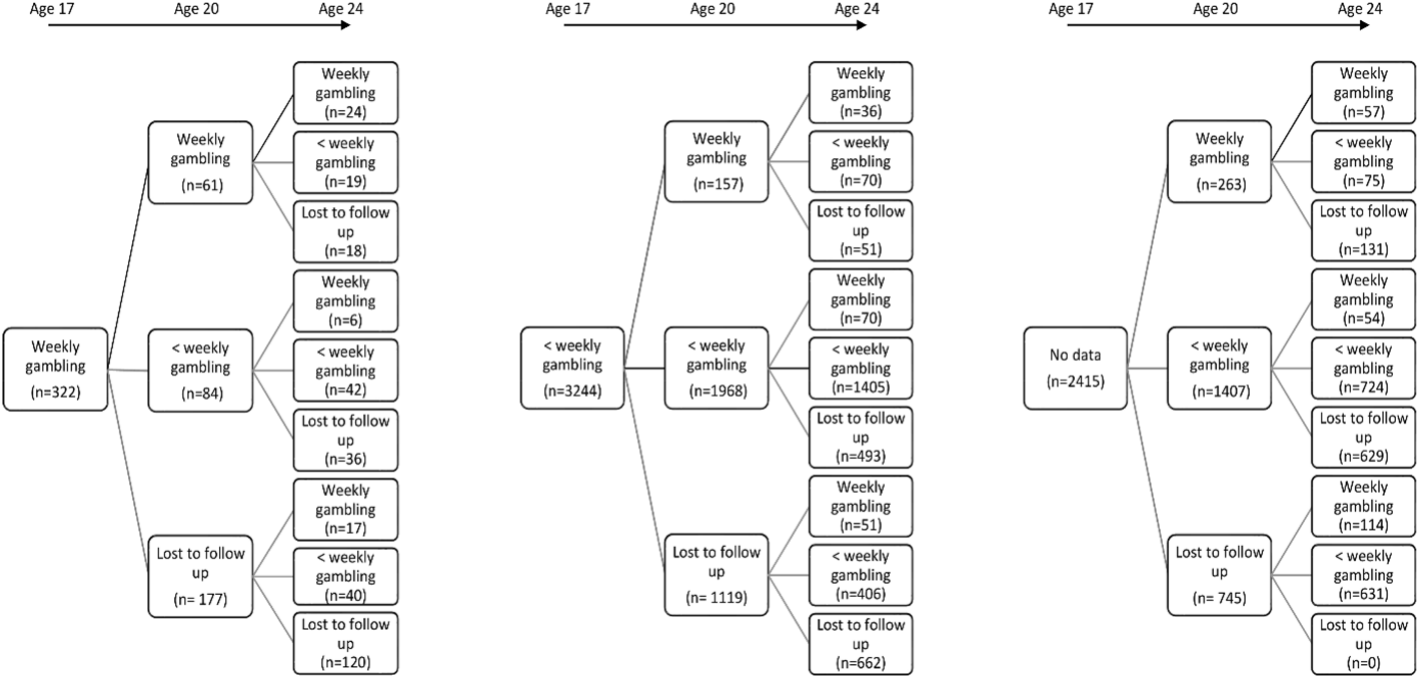
Flow of non-gamblers, occasional gamblers, and regular gamblers from 17 to 24 years

A complete case series (n = 1672) of those who completed all three gambling surveys was used to describe the gambling status of individuals across time. However, due to the amount of missing data among individual, parental and SES variables examined, detailed analyses using multiple variables on complete cases was not possible without biased estimates and loss of power. Analyses therefore proceeded by examining the three age groups separately, being aware that some individuals had repeated data points across time. However, as Fig. 1 shows, there was a substantial influx of new participants that did not answer the survey at age 17 years but did so at age 20 and 24 years.

Data were analysed in STATA v.15. (StataCorp. 2017). Univariable analyses on child, parental, and SES antecedents to gambling were conducted on all available data using chi-square tests or ANOVAs. Those variables associated at *p* < 0.05 with gambling at different ages were taken forward to the multivariable models, and are detailed in the supplementary material. Due to the number of antecedents analysed, the missing data on antecedents and the loss to follow-up, multivariable analyses were not possible without multiple imputation. Multiple imputation using 50 imputations and chained equations method were performed utilizing the mi impute command in STATA. Variables included in the imputation model were all of those included in the final regression models (many of which predicted missingness in the other variables). Earlier and later measures of some of the predictor variables as auxiliary variables were also included. Data were imputed up to the number of participants out of the total cohort that answered at least one of the three gambling surveys (N = 5981). More details including a table of the percentage of cases that were imputed for all variables in the analyses are provided in supplementary Methods 2.

Using the imputed data set, multivariable analyses of the impact of child, parental and SES variables were carried out using multinomial logistic regressions for the three time points separately in a stepwise fashion: (1) impact of each child variable adjusted for all other child variables, (2) all child variables adjusted for all parental variables, (3) all child and parental variables adjusted for all SES variables. Given that gender had a very strong influence on gambling outcomes, all multivariable analyses were stratified by gender and these results are the ones presented. Multinomial odds ratios (ORs) and 95% confidence intervals (95% CI) are reported.

## Results

### Participants

**Fig. 2:**
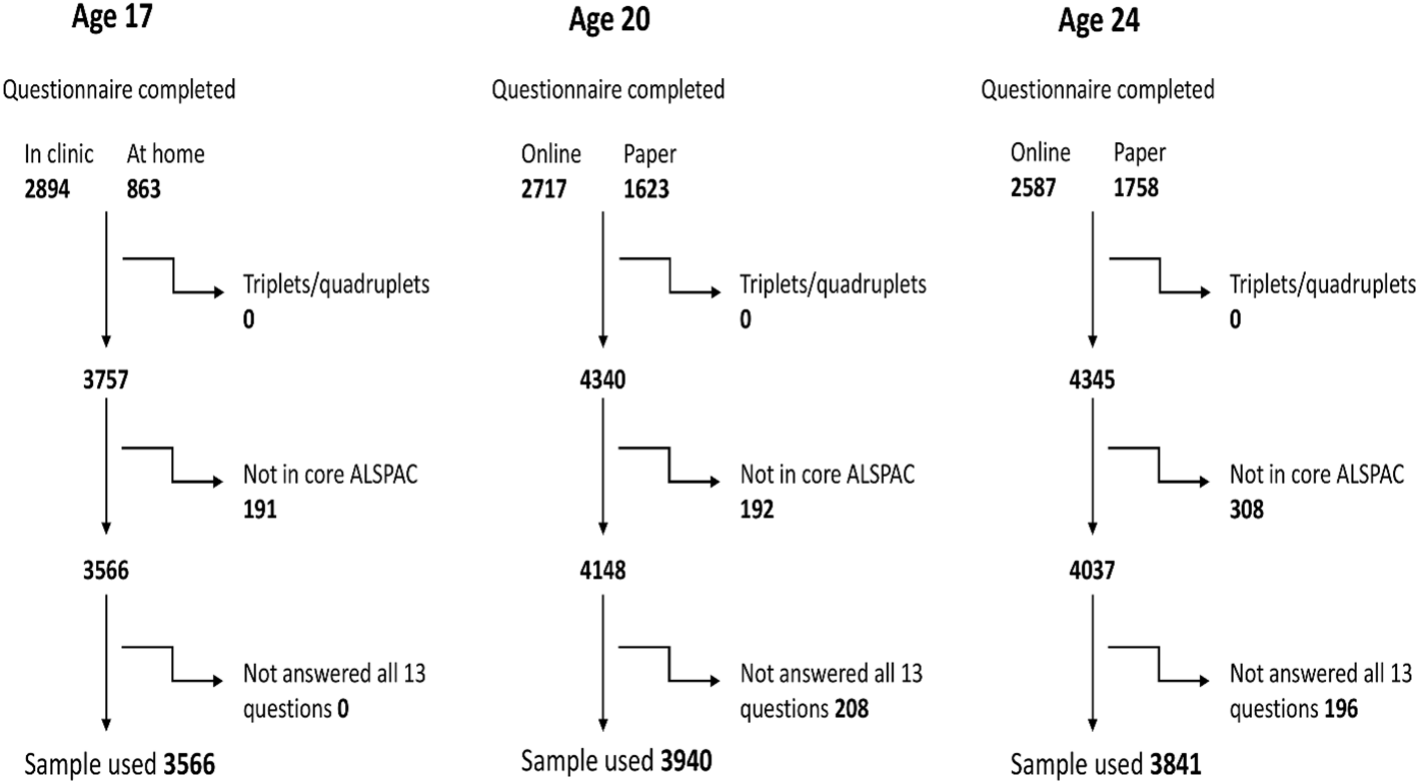
Flow-chart and exclusions at all three time points

After exclusions, 3566 participants at age 17 years, 3940 at age 20 years, and 3841 at age 24 years were included in analyses (Fig. 2). There were more females than males who completed the surveys at each time point: 58% were females at age 17 years, 61% females at age 20 years, and 65% females at age 24 years. The mean age (SD) of participants in the three sweeps were 17.8 (0.4) years, 20.9 (0.5) years, and 24.9 (0.6) years.

There was a large loss to follow-up, and only 1672 participants (1096 females and 576 males) answered all three surveys (Fig. 1). Participants who gambled at age 17 years and/or at age 20 years but were lost to follow-up at 24 years were more likely to: be male, have hyperactivity and conduct problems, have a higher sensation seeking score, be unemployed/not in education, smoke and drink alcohol weekly, have mothers with low educational qualification, more financial difficulties, and have mothers that gambled regularly when child was aged 6 years (supplementary Table 1).

### Gambling Frequency

The distribution of non-gamblers, occasional gamblers (< weekly in the past 12 months), and regular gamblers (≥ weekly in the past 12 months) across time are displayed in Table 2, using the complete case series. Non-gamblers at 17 years tended to remain in the same category at 20 and 24 years, with about a third becoming occasional gamblers, and very few were regular gamblers at 24 years. Seventy percent of occasional gamblers at 17 years were still gambling occasionally at 20 and 24 years, with 23% stopping gambling and only 7% becoming regular gamblers at 24 years. Regular gamblers at 17 years continued gambling, with 56% being regular gamblers and 37% occasional gamblers at 24 years. Overall, the variation between categories was less between 20 and 24 years than between 17 and 20 years.

**Table 2:** Distribution of non-gamblers, occasional gamblers and regular gamblers by age (complete case series)

N = 1672 complete cases	Non-gambler at 17			Occasional (< weekly) gambling at 17			Regular (> weekly) gambling at 17		
	Non-gambler at 20	Occasional at 20	Regular at 20	Non-gambler at 20	Occasional at 20	Regular at 20	Non-gambler at 20	Occasional at 20	Regular at 20
Non-gambler at 24	302 (69.3%)	118 (33.2%)	7 (17.5%)	87 (55.1%)	120 (22.8%)	4 (6.1%)	5 (55.6%)	4 (10.3%)	3 (7.0%)
Occasional at 24	126 (28.9%)	218 (61.4%)	22 (55.0%)	67 (42.4%)	367 (69.8%)	37 (56.1%)	4 (44.4%)	29 (74.4%)	16 (37.2%)
Regular at 24	8 (1.8%)	19 (5.4%)	11 (27.5%)	4 (2.5%)	39 (7.4%)	25 (37.9%)		6 (15.4%)	24 (55.8%)
Total N	436	355	40	158	526	66	9	39	43

Table 3 shows the distribution of gambling frequency by gender, utilising all available data as numbers were too small in the complete case series. The proportion of young people who reported engaging in some form of gambling at least weekly increased from 9% at 17 years to 12.2% at 20 years and reduced to 11.2% at 24 years. The increase from 17 to 20 years and then decrease at 24 years was found in both males and females, but regular gambling was strongly male-dominated in all three age groups (Table 3).

**Table 3:** Distribution of non-gamblers, occasional gamblers and regular gamblers by gender (imputed data set)

	Non-gamblers	Occasional (< weekly) Gamblers	Regular (> = weekly) Gamblers
17 years			
Males (N = 1505)	588 (39.1%)	715 (47.5%)	202 (13.4%)
Females (N = 2061)	1044 (50.7%)	897 (43.5%)	120 (5.8%)
Total (N = 3566)	1632 (45.8%)	1612 (45.2%)	322 (9.0%)
20 years			
Males (N = 1555)	401 (25.8%)	868 (55.8%)	286 (18.4%)
Females (N = 2385)	858 (36.0%)	1332 (55.8%)	195 (8.2%)
Total (N = 3940)	1259 (32.0%)	2200 (55.8%)	481 (12.2%)
24 years			
Males (N = 1362)	388 (28.5%)	740 (54.3%)	234 (17.2%)
Females (N = 2479)	904 (36.5%)	1380 (55.7%)	195 (7.9%)
Total (N = 3841)	1292 (33.6%)	2120 (55.2%)	429 (11.2%)

#### Gambling Activity Amongst Regular Gamblers

**Fig. 3:**
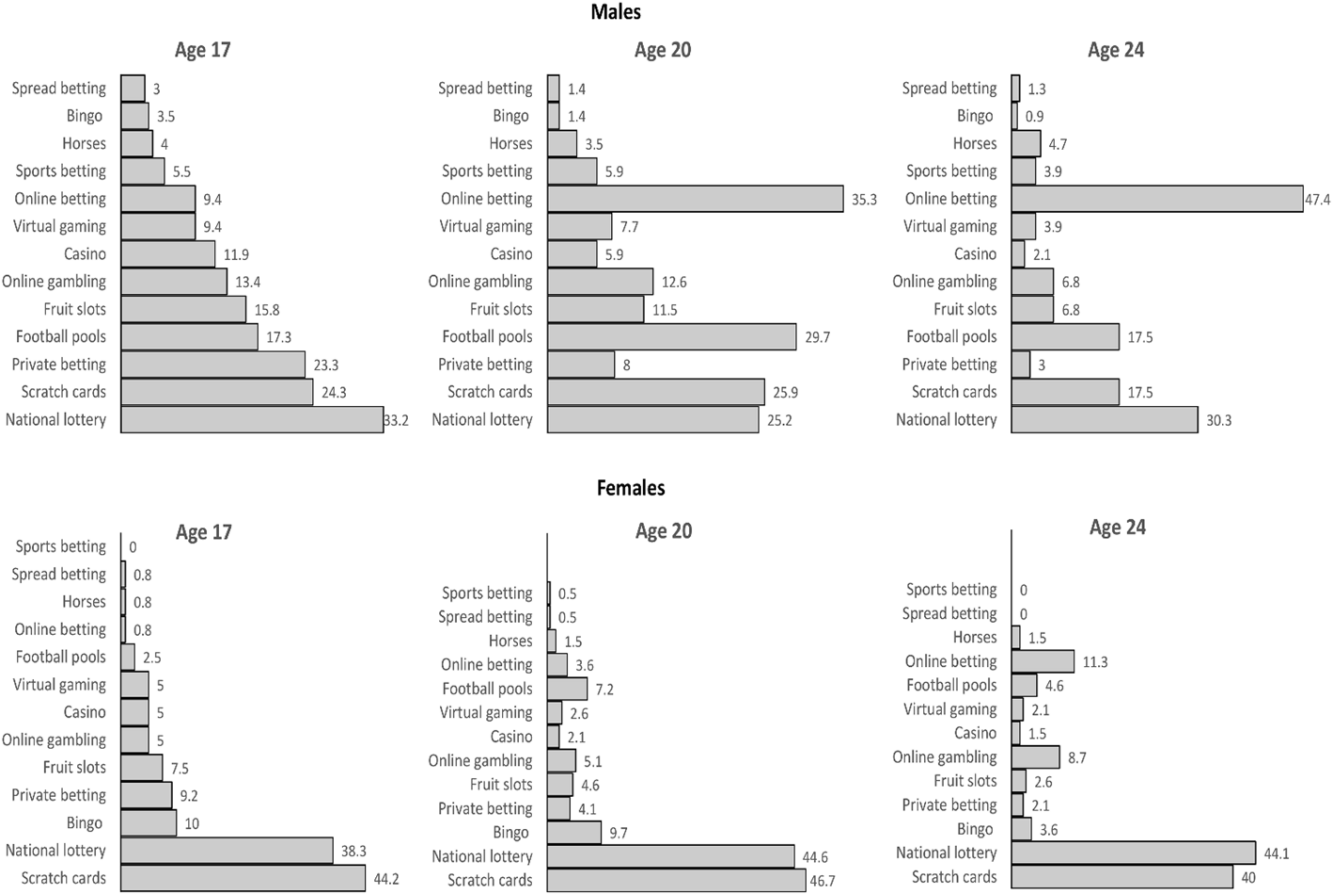
Gambling activities undertaken by regular gamblers amongst males and females

Figure 3 illustrates the types of gambling undertaken by regular gamblers at different ages, separated by gender. At age 17 years, the most commonly reported gambling activities amongst those that gambled regularly were playing scratchcards and lottery games for both males and females, but more so in females than males. Amongst the other gambling activities, private betting, football pools, slot machines, and online gambling were common in males but not so frequent in females. Females on the other hand played more bingo than males. The proportion of females gambling on scratchcards and lottery games remained relatively constant across all three time-points whereas the proportion of males playing scratchcards, in particular, dropped at age 24 years. Casino gambling actually decreased between 17 and 24 years, but betting on horseracing increased slightly. Online betting increased substantially in both males and females as they aged, but this increase was much more apparent in males and by the age of 24 years, nearly 50% of all activities constituted online betting in males compared to 11% for females (Fig. 3).

### Univariable Analyses

#### Child Antecedents

Regular gamblers were compared with occasional gamblers and those that did not gamble at all. Compared to occasional gamblers and non-gamblers, regular gamblers at age 17 years were more likely to be male, have a low IQ, prefer playing videogames with friends, a childhood history of hyperactivity and conduct problems, high scores on sensation-seeking, and higher external locus of control scores. They were also less likely to be in education or employment. Compared to the rest of the sample, regular gamblers were at least twice as likely to smoke cigarettes daily and to drink alcohol weekly. Stressful life events and diagnosed depression did not differ between regular gamblers and the rest (supplementary Table 2). Apart from hyperactivity and conduct problems, which were no different between regular gamblers and the rest at age 20 years, the remaining associations mirrored those at age 17 years (supplementary Table 3). At 24 years, regular gambling was associated with being male, low IQ, playing videogames, and not being in education or employment. Regular gamblers used social media frequently, smoked more cigarettes, and had higher levels of alcohol use (supplementary Table 4).

#### Parental/SES Antecedents

Univariable analyses showed that at all three time points, children from younger mothers, mothers with low education level, mothers who struggle financially, and parents who gamble regularly were more likely to participate in regular gambling themselves. Maternal and paternal depression did not affect gambling behavior in the offspring. Children living in council houses (a form of British social housing built by local authorities) and in overcrowded houses with large number of children per room were also more likely to gamble regularly compared to the rest (supplementary Tables 5–7).

### Multi-variable Analyses

All of the following results are from the imputed data set stratified by gender. The associations of occasional and regular gambling for males and females at all three ages, following adjustment for child variables only, are shown in supplementary Tables 8–10. The models presented in Table 4 were created using those child variables that remained significantly associated with either occasional or regular gambling after adjustment for all other child variables, which were then adjusted for parental and SES variables. Individual variables that remained associated with regular gambling after adjustment for all other child variables were lower childhood IQ in both males and females aged 17 and 20 years, higher external locus of control in both males and females aged 17 years and females only aged 20 and 24 years, weekly smoking in both males and females at all ages, harmful alcohol consumption in males aged 20 years, teenage videogame playing in females aged 20 years, and frequent social media use in both males and females aged 24 years.

**Table 4:** Multivariable models using imputed data set (N = 5981)

	Model 1 OR (95% CI)^a^	Model 2 OR (95% CI)^a^	Model 3 OR (95% CI)^a^
*Age 17*			
Males			
IQ at 8 years			
Bottom quartile	**2.37 (1.56, 3.59)**	**1.95 (1.27, 3.01)**	**2.01 (1.27, 3.17)**
Locus of control at 16.5 years			
> Median [external]	**2.22 (1.57, 3.15)**	**1.97 (1.38, 2.81)**	**2.00 (1.40, 2.85)**
Sensation seeking at 17 years	1.02 (0.99, 1.05)^b^	1.03 (1.00, 1.06)	**1.03 (1.00, 1.07)**
Smoking cigarettes at 16.5 years			
Tried	**1.61 (1.02, 2.54)**	**1.89 (1.19, 2.98)**	**1.86 (1.16, 2.96)**
< Weekly	1.72 (0.80, 3.70)	2.12 (0.97, 4.64)	2.15 (0.97, 4.76)
≥ Weekly	**2.10 (1.13, 3.93)**	**2.57 (1.43, 4.63)**	**2.57 (1.41, 4.67)**
Females			
IQ at 8 years			
Bottom quartile	**1.74 (1.09, 2.79)**	1.30 (0.80, 2.12)	1.26 (0.77, 2.06)
Locus of control at 16.5 years			
> Median [external]	**2.10 (1.37, 3.20)**	**1.86 (1.20, 2.87)**	**1.82 (1.17, 2.82)**
Smoking cigarettes at 16.5 years			
Tried	1.53 (0.93, 2.50)	1.56 (0.98, 2.50)	1.57 (0.98, 2.54)
< Weekly	1.64 (0.72, 3.73)	1.64 (0.73, 3.69)	1.64 (0.73, 3.69)
≥ Weekly	**3.45 (1.92, 6.21)**	**3.40 (2.01, 5.78)**	**3.36 (1.97, 5.71)**
*Age 20*			
Males			
IQ at 8 years			
Bottom quartile	**2.30 (1.49, 3.57)**	**1.88 (1.18, 2.98)**	**1.82 (1.14, 2.91)**
Sensation seeking at 17 years	1.02 (0.99, 1.05)^b^	**1.03 (1.01, 1.06)**	**1.04 (1.01, 1.07)**
Smoking cigarettes at 20 years			
≥ Weekly	**1.93 (1.26, 2.95)**	**1.72 (1.13, 2.62)**	**1.71 (1.11, 2.62)**
Alcohol use at 20 years			
Hazardous	**2.14 (1.53, 2.98)**	**2.34 (1.66, 3.28)**	**2.33 (1.65, 3.31)**
Harmful	**4.31 (2.59, 7.18)**	**5.20 (3.04, 8.91)**	**5.33 (3.08, 9.22)**
Females			
IQ at 8 years			
Bottom quartile	**1.85 (1.18, 2.91)**	1.26 (0.79, 2.03)	1.15 (0.71, 1.86)
Playing videogames with friends at			
13/14 years	**1.46 (1.01, 2.13)**	**1.48 (1.00, 2.18)**	1.46 (0.98, 2.17)
Locus of control at 16.5 years			
> Median [external]	**1.58 (1.12, 2.24)**	1.24 (0.87, 1.78)	1.18 (0.82, 1.70)
Smoking cigarettes at 20 years			
≥ Weekly	**2.40 (1.63, 3.53)**	**2.11 (1.44, 3.11)**	**1.99 (1.35, 2.95)**
Alcohol use at 20 years			
Hazardous	1.25 (0.88, 1.77)^b^	1.43 (0.99, 2.06)	**1.52 (1.05, 2.20)**
Harmful	1.32 (0.75, 2.31)^b^	1.65 (0.93, 2.92)	**1.80 (1.01, 3.22)**
Males			
Smoking cigarettes at 23 years			
≥ Weekly	**2.38 (1.49, 3.79)**	**2.33 (1.47, 3.68)**	**2.37 (1.49, 3.78)**
Social media use at 24 years			
2–10 times/day	**1.69 (1.03, 2.75)**	**1.76 (1.05, 2.93)**	**1.80 (1.07, 3.02)**
> 10 times/day	**2.74 (1.63, 4.61)**	**2.94 (1.73, 4.99)**	**3.05 (1.78, 5.21)**
*Age 24*			
Females			
Locus of control at 16.5 years			
> Median [external]	**1.43 (1.00, 2.06)**	1.17 (0.81, 1.68)	1.09 (0.75, 1.60)
Smoking cigarettes at 23 years			
≥ Weekly	**3.00 (1.88, 4.79)**	**2.68 (1.71, 4.19)**	**2.51 (1.58, 4.00)**
Social media use at 24 years			
2–10 times/day	2.08 (0.97, 4.45)	1.83 (0.87, 3.84)	1.92 (0.89, 4.13)
> 10 times/day	**2.97 (1.37, 6.45)**	**2.61 (1.23, 5.55)**	**2.80 (1.29, 6.09)**

Multinomial odds ratios and 95% confidence intervals (CI) refer to ***regular*** gambling versus no gambling

The child variables significant after adjustment for all other child variables were adjusted for parental and socioeconomic variables for males (N = 2486) and females (N = 3495) at 17, 20 and 24 years

Those significant after full adjustment at one or more time points are shown in bold

^a^Model 1: adjusted for other child variables; Model 2: model 1 + adjusted for maternal age, maternal education, maternal gambling at 6 and 18 years, paternal gambling at 6 years; Model 3: model 2 + adjusted for crowding index, financial difficulties, housing status and Index of Multiple Deprivation

^b^ These variables were kept in as occasional gambling was significantly associated with them (shown in Supplementary Table 11)

Many of the associations with regular gambling and child variables remained after adjustment for parental and SES variables. Several associations seen in females attenuated after adjustment: lower childhood IQ and regular gambling at age 17 and 20 years, external locus of control at age 20 and 24 years, and previous videogame playing at age 20 years. The effect of sensation seeking at age 17 and 20 years in males appeared to get stronger after adjustment. Likewise, the association with alcohol consumption in females aged 20 years became stronger after adjustment (Table 4).

The individual effects of parental and SES variables on occasional and regular gambling are shown in supplementary Table 12. Variables that remained strongly associated with regular gambling after full adjustment were: lower maternal education level for both males and females at all ages, maternal gambling for males aged 20 and 24 years, and females aged 24 years, paternal gambling for males at all ages but females only at age 20 years, and living in council/housing association accommodation strongly increased likelihood of regular gambling in females aged 24 years.

The final models for regular gambling are presented in Table 5, using only those antecedents (individual, parental and SES) which were significant after full adjustment at one or more of the three time points. A similar summary table for occasional gambling is provided in supplementary Table 13.

**Table 5:** Summary table of fully adjusted multinomial odds ratios for regular (weekly) gambling in males and females at each of the three time points

Fully adjusted ORs (95% CI)	Males			Females		
	Age 17 years	Age 20 years	Age 24 years	Age 17 years	Age 20 years	Age 24 years
IQ at 8 years						
Bottom quartile	**2.01 (1.27, 3.17)**	**1.82 (1.14, 2.91)**				
Locus of control at 16.5 years						
> Median [external]	**2.00 (1.40, 2.85)**			**1.82 (1.17, 2.82)**		
Sensation seeking at 17 years	**1.03 (1.00, 1.07)**	**1.04 (1.01, 1.07)**				
Smoking cigarettes at 16.5 years						
Tried	**1.86 (1.16, 2.96)**			1.57 (0.98, 2.54)		
< Weekly	2.15 (0.97, 4.76)			1.64 (0.73, 3.69)		
≥ Weekly	**2.57 (1.41, 4.67)**			**3.36 (1.97, 5.71)**		
Smoking cigarettes at 21 years						
≥ Weekly		**1.71 (1.11, 2.62)**			**1.99 (1.35, 2.95)**	
Alcohol use at 21 years						
Hazardous		**2.33 (1.65, 3.31)**			**1.52 (1.05, 2.20)**	
Harmful		**5.33 (3.08, 9.22)**			**1.80 (1.01, 3.22)**	
Smoking cigarettes at 23 years						
≥ Weekly			**2.37 (1.49, 3.78)**			**2.51 (1.58, 4.00)**
Social media use at 24 years						
2–10 times/day			**1.80 (1.07, 3.02)**			1.92 (0.89, 4.13)
> 10 times/day			**3.05 (1.78, 5.21)**			**2.80 (1.29, 6.09)**
Maternal education pregnancy						
Degree higher than A level	**0.27 (0.14, 0.53)**	**0.34 (0.17, 0.68)**	**0.38 (0.20, 0.74)**	**0.15 (0.05, 0.47)**	**0.18 (0.08, 0.40)**	**0.37 (0.18, 0.77)**
Maternal gambling child age 6 years						
< Weekly		**1.88 (1.20, 2.93)**	**1.78 (1.11, 2.86)**			1.51 (0.90, 2.52)
≥ Weekly		**2.06 (1.31, 3.26)**	**2.58 (1.70, 3.91)**			**2.43 (1.50, 3.93)**
Paternal gambling child age 6 years						
< Weekly	1.53 (0.84, 2.81)	**1.67 (1.00, 2.78)**	1.59 (0.95, 2.66)		1.33 (0.75, 2.36)	
≥ Weekly	**2.19 (1.12, 4.29)**	**2.23 (1.25, 3.98)**	**1.84 (1.09, 3.10)**		**2.23 (1.20, 4.12)**	
Mother’s gambling child age 18 years						
No problem gambler	**1.51 (1.01, 2.26)**					
Low–high risk gambler	1.74 (0.60, 5.10)					
Housing child age 18 years						
Council/housing association						**2.60 (1.31, 5.14)**

Those significant after full adjustment at one or more time points are shown in bold. The blank entries are either non-significant or not measured at that age

## Discussion

The ALSPAC Gambling Study, utilising an existing cohort of young people, demonstrated that overall rates of gambling increased between 17 and 20 years but varied little thereafter. The first study hypothesis was therefore only partially upheld. Online gambling increased the most, which may reflect the widening use of the internet during the study period (2009–2017). Gambling on horseraces also increased from 17 to 20 years and 24 years.

Between 9 and 12% of young people were regular weekly gamblers. These were more likely to be males, to come from families in which parents gambled, and living in more deprived circumstances (residing in social/public housing aged 18 years). Individual factors consistently associated with regular gambling were low IQ, having a high external locus of control, and high sensation seeking scores in males. Strong associations were also found with smoking cigarettes, alcohol consumption, and high social media usage. Parental factors associated with regular gambling in young people were past and current gambling, and low maternal educational attainment. The second hypothesis, that regular gambling would be associated with both family factors and individual developmental traits, was therefore supported by the findings.

Although it was disappointing that the lack of variance in gambling behavior between 17 and 24 years did not permit longitudinal trajectory modelling, this is an important finding which confirms that gambling habits in young adulthood appear to be established in late adolescence. The Gambling Commission’s (2018) report on young people and gambling found that, 39% of 11–16 year olds had spent their own money on gambling over the previous year. A Canadian study (Auger et al. 2010) reported a median age of gambling onset of 17 years. In ALSPAC, the regular gamblers appeared to be established by age 20 years.

The rise in use of online gambling in young adults is consistent with the Gambling Commission’s (2018) study which found that 13% of 11–16 year olds had played gambling-style games online and 31% had bought loot boxes within a videogame or app, as well as the findings within the contemporary online gambling literature more generally (e.g., Canale et al. 2016; Lopez-Gonzalez and Griffiths 2018). Gambling and betting online showed the largest increase from the ages of 17 to 24 years. Not only is this likely to be a function of the increasing convergence between various online activities (particularly gambling and gaming), but also because the past decade has seen a large increase in sports betting online (Lopez-Gonzalez et al 2017, 2018, 2019; Lopez-Gonzalez and Griffiths 2018), particularly in the form of in-play betting where individuals can now place bets during the game itself (Killick and Griffiths 2019). Online in-play betting is now heavily advertised in the UK and more engaged in by males than females (Lopez-Gonzalez et al. 2018). The increase seen in in-game sports betting was probably related to increased legal access after 18 years, and greater disposable income. The rise in popularity of this one specific form of gambling among males may also be a major contributory factor to the increase in betting online among males from the ages of 17 to 24 years.

Individual factors found to be associated with regular gambling from 17–24 years were largely consistent with the literature, with recognised correlations with low IQ (Rai et al. 2014) hyperactivity and impulsivity (Breyer et al. 2009; Faregh and Deverensky 2011), and sensation seeking (Nower et al. 2004). The associations of regular gambling with high external locus of control (feeling low personal control over one’s life) were consistent across both genders. A high external locus of control has been associated with other potentially addictive behaviours, including the playing of videogames (Lloyd et al. 2019). However, other studies of gambling in this age group (e.g., Moore and Ohtsuka 1999) have found that illusion of control and *internal* control over gambling predicted gambling frequency and problem gambling. This difference may be related to the type of gambling activity, as the commonest activities in the ALSPAC were playing the lottery and scratchcards. Lester (1980) showed that undergraduates with a belief in an external locus of control gambled more at games in which luck played a part and less at games in which skill and judgment played a part.

Although there were no gender differences among those who gambled occasionally (mainly on the lottery and scratchcards), there appeared to be a strong association of regular gambling with being male. Regular gamblers in the ALSPAC were boys who had also been players of videogames at 14 years, and the rise in online gambling seen at 20 and 24 years was almost exclusively seen in young men. This association has been widely reported in literature reviews of both adults and adolescents (e.g., Calado et al. 2017; Calado and Griffiths 2016). Previous reviews have suggested such gender differences may be due to sex role socialisation, sub-cultural features of gambling, personality differences, motivational gender differences, genetic differences, and differences in psychiatric comorbidity, among others (e.g., Delfabbro 2000; Holdsworth et al. 2012; Martins et al. 2002; Merkouris, et al. 2016). Derevensky and Gilbeau (2015) reviewed the many issues and correlates impacting on adolescent gambling and problem gambling and highlighted the importance of early exposure to gambling in the family home, and the use of the internet. Calado et al. (2017) argued that the emergence of gambling within new technologies in the family home enables parents not only to gamble in the presence of their children but also turns gambling into a potential family activity. The data in the present study suggest that as young men gain more independence, the widespread availability of gambling apps on smartphones, combined with immature self-regulation, impulsivity, and an external locus of control, increases the frequency of gambling.

The most important family factors associated with regular gambling in the young people were parental gambling and mothers’ educational level. Parental gambling behaviour was strongly associated with their children’s regular gambling, with mother’s gambling frequency having the strongest effect after adjustment. Vachon et al. (2004) reported that adolescent gambling frequency was related to both parents' gambling frequency and problems, but that adolescent gambling problems were only associated with fathers' severity of gambling problems. Mothers’ educational level remained a significant factor for regular gambling in both their male and female children, whereas the effect of SES attenuated after adjustment (see Barnes et al. 2005).

The strength of the present study was its use of the large ALSPAC cohort, which has collected a wealth of data for over 25 years. When this cohort was initiated in 1991, it was representative of a whole community and it covered a range of environments from inner city to semi-rural in one geographical area. The ALSPAC has also collected a diverse range of psychological and physical measures from both the children and their families. Parental gambling activity was collected by self-report independently from their children’s report. Gambling activity at 17, 20, and 24 years was self-reported by the young people, not by their parents, and a wealth of background information was available on these families. The novel findings of this study are (1) the demonstration that gambling frequency increases between 17 and 20, but remains stable until 24 years, (2) the steady increase in the use of the internet for gambling during the study, (3) the association between regular gambling and having an external locus of control, and (4) the high social media use reported by those males and females who gambled regularly.

The main limitation of this research is the missing data, with only 62% of the whole cohort completing the gambling station in the 17 + years research clinic or completing the online surveys. Non-responders to the gambling surveys, when compared to responders, were more likely to be male and from more deprived social backgrounds, with mothers with lower educational levels. Multiple imputation techniques were used to minimise the bias from attrition, but the analyses probably underestimated the prevalence of regular gambling. There was also a significant gender bias, with the final sample comprising 58% females. As males were more likely to engage in all types of gambling activity, this gender bias in reporting will have resulted in an under-estimate of gambling prevalence and associated characteristics. The ALSPAC sample also was predominately white, with too few young people from Black or Asian heritage to permit any valid comparisons of gambling behaviour in different ethnic groups. All the gambling data were self-reported, and therefore were subject to many biases including social desirability and memory recall. Although a cohort with data at three time-points was used, the data did not allow a full utilization of the longitudinal nature of the study, and so causal inferences cannot be made.

## Conclusions

Although more than half the sample of young people aged 17 to 24 years participated in gambling, most engaged in occasional activity with lottery or scratchcards, and there was little overall change in gambling behaviour between these ages. Many young people had tried different forms of gambling between 17 and 24 years, but one activity showing a large increase over this age range was online gambling and betting. Between 6–8% of females and 13–18% of males were regular weekly gamblers, and patterns of regular gambling appeared to be set by the age of 20 years. Regular gamblers were predominantly males, who were increasingly gambling online, with clear associations with smoking cigarettes, alcohol consumption, and high social media usage.

## Electronic supplementary material

Below is the link to the electronic supplementary material.

Supplementary file1 (DOCX 92 kb)
