# Supplementary material for: Gambling in Young Adults Aged 17–24 Years: A Population-Based Study
Source: J Gambl Stud. 2020 Apr 18;36(3):747–66. doi: 10.1007/s10899-020-09948-z (PMC7395026; doi:10.1007/s10899-020-09948-z)
Supplement: Supplementary file 1 — Supplementary file1 (DOCX 92 kb) [file 10899_2020_9948_MOESM1_ESM.docx]

**Supplementary Material Contents:**

**Supplementary methods 1.** Description of variables used in the analyses**.**

**Supplementary methods 2.** Missing data and multiple imputation methods.

**Supplementary table 1**. Demographics of participants lost to follow up.

**Supplementary table 2**. Univariable results on the association between child antecedents and gambling activity at age 17.

**Supplementary table 3**. Univariable results on the association between child antecedents and gambling activity at age 20**.**

**Supplementary table 4**. Univariable results on the association between child antecedents and gambling activity at age 24.

**Supplementary table 5**. Univariable results on the association between parental and socioeconomic antecedents and gambling activity at age 17.

**Supplementary table 6**. Univariable results on the association between parental and socioeconomic antecedents and gambling activity at age 20**.**

**Supplementary table 7**. Univariable results on the association between parental and socioeconomic antecedents and gambling activity at age 24.

**Supplementary table 8**. Unadjusted and adjusted multinomial odds ratios for child variables only at age 17.

**Supplementary table 9**. Unadjusted and adjusted multinomial odds ratios for child variables only at age 20.

**Supplementary table 10**. Unadjusted and adjusted multinomial odds ratios for child variables only at age 24.

**Supplementary table 11**. Adjusted multinomial odds ratios for parental and socioeconomic antecedents on occasional gambling at age 17, 20 and 24.

**Supplementary table 12**. Individual effects of parental and socioeconomic antecedents on occasional and regular gambling at age 17, 20 and 24.

**Supplementary table 13** Summary table of fully adjusted multinomial odds ratios for occasional gambling in males and females at age 17, 20 and 24.

**Supplementary methods 1. Description of variables used in the analyses.**

| Variable | Age (years) | Description |
| --- | --- | --- |
| ***Child antecedents*** |  |  |
| IQ | 8 | A short version of the WISC III^1^ applied by trained psychologists in research clinic was used. We used the total IQ (verbal + performance) and compared the % of people in the bottom quartile to the rest. |
| Video games | 13 | Teenagers were asked whether they chose to play video games with other children instead of other activities. This was used as a binary yes/no variable. |
| Hyperactivity and conduct problems | 16.5 | Measured using the Strengths and Difficulties Questionnaire (SDQ). Scores were entered as binary variables based on cut-offs for ‘abnormal’ scoring on each SDQ subscale as suggested by Goodman.^2^ |
| Locus of control | 16.5 | Calculated summing the answers on a 12 item Nowicki-Strickland Locus Of Control Scale^3^. People with a lower score believe that an outcome is largely contingent upon their own behaviour and are having a more *internal* locus of control, whereas those with a higher score believe that luck, fate, chance or powerful others largely determine an outcome are more *external*. Scores greater than the median were labelled external and less than or equal to the median were labelled internal. |
| Sensation seeking | 17 | A total sensation seeking score (novelty subscale + intensity subscale) was measured using the Arnett Sensation Seeking Scale^4^. A higher score indicates a higher tendency to pursue sensory pleasure and excitement. |
| Stressful life events | 16 | Teenagers were asked in a series of questions whether they had experienced major stressful events such as death of a family member, pregnancy, arrival of siblings etc since the age of 12. A summed continuous score was used for analyses. |
| Education/employment status | 17, 20 | Participants were asked whether they were in education or employment (full or part-time). This was used as a binary yes/no variable. |
| Depression | 17 | An ICD-10 diagnosis of depression (yes/no) established using the CIS-R completed in n a research clinic was used. |
| Smoking | 16.5, 20, 23 | Participants were asked about cigarette smoking habits. We used the % of weekly smokers compared to those that did not smoke weekly. |
| Alcohol consumption | 16, 20, 23 | Alcohol consumption was measured slightly differently at each time point. At age 16, we used the % of weekly alcohol intake compared to the rest. At age 20, we used the % of harmful alcohol use compared to the rest and at age 23, we used the DSM4 criteria of alcohol abuse (yes/no). |
| Social media use | 24 | Measured as the frequency of using social media. We compared the % using it >10 times/day to those that used it less frequently. |
| ***Parental antecedents*** |  |  |
| Maternal age | At birth | We used the % of women above or below the mean age of all women at the birth of their child. |
| Maternal education | 32 weeks gest. | Measured as the highest education level the mother held. It was classified as CSE (Certificate of Secondary Education)/none, Vocational, O level, A level, Degree. We compared the proportion of mothers with a degree compared to those with levels below a degree. |
| Maternal and partner depression | Child aged 12 | Mother and mother’s partner were asked if they had experienced depression (yes/no) in the past 2 years. |
| Maternal/paternal gambling | Child aged 6, 18 | Questionnaire data on maternal and paternal gambling were collected using the South Oaks Gambling Screen^5^ when the children were aged 6 years. We compared weekly parental gambling to the rest. Maternal gambling data was also collected using the Canadian Problem Gambling Index^6^ where mothers were classified into non-gamblers, no-problem gamblers, low risk gamblers, moderate risk gamblers and problem gamblers. |
| ***Socioeconomic antecedents*** |  |  |
| Crowding index | 8 weeks gest. | Calculated by dividing the number of people in the household by the number of rooms and categorising as [0, 0.5], [0.5, 0.75], [0.75, 1], [> 1]. The higher the number, the more crowded a household. We compared the proportion with an index of >1 to the rest. |
| Financial difficulties | 32 weeks gest. | A numerical score was created from five questions about how difficult the mothers found affording certain items. The higher the score the more financial difficulties. We compared the top tertile with the rest. |
| Index of multiple deprivation | Child aged 11 | IMD is created from census data on 7 socio-economic domains: income; employment; health and disability; education, skills and training; barriers to housing and services; living environment; and crime. The IMD was based on the address of the family when the child was 11 years old; the highest IMD quintile indicates the greatest social deprivation. |
| Housing | Child aged 18 | Mothers were asked about their housing situation. We used a derived variable comparing those living in social housing (council/housing association) to those who owned their own or private renting. |

**References**

1. Wechsler D, Golombok S, Rust J. 1992. WISC-IIIUK: Wechsler Intelligence Scale for Children. Sidcup, UK: Psychological Corporation.
2. Goodman A, Goodman R. 2009. Strengths and difficulties questionnaire as a dimensional measure of child mental health. *J Am Acad Child Adolesc Psychiatry*; **48**: 400-403.
3. Nowicki, S. & Strickland, BR. 1973. A locus of control scale for children. Journal of Consulting and Clinical Psychology; **40**: 148-154.
4. Arnett, J. 1994. Sensation Seeking: A new conceptualization and a new scale.
5. Lesieur, HR. & Blume, SB. 1987. The South Oaks Gambling Screen (SOGS): a new instrument for the identification of pathological gamblers. *Am J Psychiatry*; **144**:1184-1188.
6. Ferris, J. & Wynne, H. 2001. The Canadian problem gambling index: Final report. Submitted for the Canadian Centre on Substance Abuse. *Personality and Individual Differences;* **16**:289-296.
7. Patton G, Coffey C, Posterino M, Carlin J, Wolfe R, Bowes G. 1999. A computerised screening instrument for adolescent depression: population-based validation and application to a two-phase case-control study. *Social Psychiatry and Psychiatric Epidemiology*; **34**:166–172.

**Supplementary methods 2. Missing data and multiple imputation methods.**

Missing data is a widespread problem in longitudinal cohort studies such as ALSPAC. At age 17, 72% of those invited to the clinic completed the gambling questionnaire. At 20 and 24, this was reduced to 47%. And even amongst those that answered the questionnaires, up to 40% had missing data on the outcome and antecedent variables used (see table). Hence, it is likely that without taking this into account, our results would be biased. Multiple imputation is a common technique used to correct for bias introduced by loss to follow-up.^1^ We used multiple imputation by chained equations using the “mi impute” command in Stata v.15.1 (StataCorp. 2017). We imputed up to the number of participants who had answered at least one of the three gambling questionnaires (N = 5981) using 50 imputations. Imputation model diagnostics were performed using the command ‘midiagplots’ which compares the distributions of the observed, imputed, and completed values.^2^ Binary variables and categorical variables used logistic, ordinal and multinomial regression, as appropriate, specified in the mi impute command. Normally distributed variables were incorporated using linear regression in the imputation model. To identify auxiliary variables, we explored factors that were associated with missingness in our predictors and covariates and those that were highly associated with missingness (p<0.05; helping to support the assumption of “missing at random”) were included in the final imputation model, together with those included in the final regression models.

**References**

1. Sterne JA, White IR, Carlin JB, Spratt M, Royston P, Kenward MG, Wood AM, Carpenter JR. Multiple imputation for missing data in epidemiological and clinical research: potential and pitfalls. *BMJ* 2009; 38:b2393.
2. Eddings W, Marchenko Y. Diagnostics for multiple imputation in Stata. *Stata Journal* 2012; 12(3):353

Table of imputed percentage for all variables used in analyses.

| **Variables** | **Total** | **Complete** | **Imputed** | **Imputed %** |
| --- | --- | --- | --- | --- |
| Gender | 5981 | 5981 | 0 | 0.00 |
| Maternal education | 5981 | 5768 | 213 | 0.04 |
| Crowding index | 5981 | 5733 | 248 | 0.04 |
| Maternal financial difficulties | 5981 | 5618 | 363 | 0.06 |
| Maternal age at birth | 5981 | 5615 | 366 | 0.06 |
| Maternal gambling (study child age 6) | 5981 | 4993 | 988 | 0.17 |
| Index of Multiple Deprivation | 5981 | 4580 | 1401 | 0.23 |
| IQ at 8 | 5981 | 4541 | 1440 | 0.24 |
| Maternal depression (study child age 10) | 5981 | 4485 | 1496 | 0.25 |
| Computer games use (age 13) | 5981 | 4271 | 1710 | 0.29 |
| Hyperactivity at 16.5 | 5981 | 4140 | 1841 | 0.31 |
| Conduct problems at 16.5 | 5981 | 4138 | 1843 | 0.31 |
| Smoking at 17 | 5981 | 4051 | 1930 | 0.32 |
| Stressful life events at 16 | 5981 | 4049 | 1932 | 0.32 |
| Smoking at 21 | 5981 | 3999 | 1982 | 0.33 |
| Regular gambling at 21 | 5981 | 3940 | 2041 | 0.34 |
| In education/employment at 21 | 5981 | 3922 | 2059 | 0.34 |
| Social media use at 24 | 5981 | 3868 | 2113 | 0.35 |
| Depression at 23 | 5981 | 3851 | 2130 | 0.36 |
| Regular gambling at 25 | 5981 | 3841 | 2140 | 0.36 |
| Locus of control at 16 | 5981 | 3800 | 2181 | 0.36 |
| Alcohol use at 17 | 5981 | 3768 | 2213 | 0.37 |
| Alcohol use at 21 | 5981 | 3742 | 2239 | 0.37 |
| Paternal depression (study child age 10) | 5981 | 3708 | 2273 | 0.38 |
| Regular gambling at 17 | 5981 | 3566 | 2415 | 0.40 |
| In education/employment at 17 | 5981 | 3549 | 2432 | 0.41 |
| Smoking at 23 | 5981 | 3477 | 2504 | 0.42 |
| Depression at 23 | 5981 | 3458 | 2523 | 0.42 |
| Alcohol use at 23 | 5981 | 3431 | 2550 | 0.43 |
| Housing at 18 | 5981 | 3339 | 2642 | 0.44 |
| Maternal gambling (study child age 18) | 5981 | 3314 | 2667 | 0.45 |
| Paternal gambling (study child age 6) | 5981 | 2940 | 3041 | 0.51 |
| Sensation seeking at 17 | 5981 | 2858 | 3123 | 0.52 |

**Supplementary table 1. Demographics of participants lost to follow up** Imputed data set (N=5981).

| **Characteristics/confounders** | **Lost to follow up^*^**  **(N tot=2140)** | **Rest of gambling cohort**  **(N tot=3841)** | **P-value^*^** |
| --- | --- | --- | --- |
| ***YP variables*** |  |  |  |
| Gender (% male) | 52.5% | 35.5% | **<0.001** |
| IQ at 8 (% bottom quartile) | 19.3% (n=1553) | 17.2% (n=2988) | 0.08 |
| Plays computer games with other children at 13/14  (% yes) | 63.1% (n=1475) | 56.6% (n=2796) | **<0.001** |
| Hyperactivity at 16.5  (% abnormal; score 7-10) | 6.2% (n=1355) | 3.7% (n=2785) | **<0.001** |
| Conduct problems at 16.5  (% abnormal; score 4-10) | 5.7% (n=1351) | 4.1% (n=2787) | **0.02** |
| Locus of control at 16.5  (% >median [external]) | 38.8% (n=1161) | 36.1% (n=2639) | 0.11 |
| Sensation seeking at 17  (mean (SD)) | 53.2 (7.1) (n=733) | 51.7 (7.4) (n=2125) | **<0.001** |
| Stressful life events age 16  (mean (SD)) | 3.0 (2.1) (n=1241) | 2.9 (2.0) (n=2808) | 0.62 |
| YP is in education or employment age 17  (% no) | 13.7% (n=1298) | 10.1% (n=2251) | **0.001** |
| Depression at 17  (ICD-10 diagnosis = % yes) | 7.0% (n=1392) | 8.1% (n=2459) | 0.21 |
| Smoking cigarettes at 16.5  (% ≥ weekly) | 13.9% (n=1242) | 10.6% (n=2809) | **0.005** |
| Alcohol use at 16.5  (% weekly) | 17.9% (n=1155) | 14.1% (n=2613) | **0.001** |
| ***Parental/socio-economic variables*** |  |  |  |
| Maternal age at birth  (% < mean) | 37.5% (n=2004) | 34.4% (n=3611) | **0.02** |
| Maternal education pregnancy  (% with degree higher than A level) | 15.2% (n=2065) | 20.0% (n=3703) | **<0.001** |
| Crowding index pregnancy  (% >1) | 4.0% (n=2049) | 3.7% (n=3684) | 0.24 |
| Financial difficulties pregnancy  (% difficulty score >3) | 28.9% (n=2002) | 24.7% (n=3616) | **0.001** |
| Maternal depression (child age 10-12)  (% yes) | 22.0% (n=1547) | 20.5% (n=2938) | 0.23 |
| Partner’s depression (child age 10-12)  (% yes) | 18.5% (n=1283) | 18.4% (n=2425) | 0.95 |
| Index of Multiple Deprivation (child age 11)  (% most deprived quintile) | 19.4% (n=1586) | 16.8% (n=2994) | 0.06 |
| Housing child age 18  (% Council/housing association) | 4.8% (n=1077) | 5.0% (n=2262) | 0.52 |
| Maternal gambling child age 6  (% ≥ Weekly) | 42.8% (n=1720) | 38.1% (n=3273) | **<0.001** |
| Paternal gambling child age 6  (% ≥ Weekly) | 49.4% (n=951) | 47.2% (n=1989) | 0.06 |
| Mother’s gambling child age 18  (% Low-High risk gambler) | 2.4% (n=1065) | 2.2% (n=2249) | 0.09 |

^*^Has been gambling at either 17 and 20 or both but have no data at 24. P-values stem from Chi-square tests or t-tests.

**Supplementary table 2**. **Univariable results on the associations between child antecedents and gambling activity at age 17.**

|  | **Age 17** | | | |
| --- | --- | --- | --- | --- |
| **Variables** | **No gambling**  **(N tot=1632)** | **< Weekly gambling (occasional)**  **(N tot=1612)** | **≥ Weekly gambling (regular)**  **(N tot=322)** | **P-value^*^** |
| Gender (% male) | 36.0% | 44.4% | 62.7% | **<0.001** |
| IQ at 8 (% bottom quartile) | 14.5%  N=1383 | 14.7%  N=1349 | 29.6%  N=253 | **<0.001** |
| Plays video games with other children at 13/14 (% yes) | 55.8%  N=1372 | 62.2%  N=1322 | 68.8%  N=253 | **<0.001** |
| Hyperactivity at 16.5  (% abnormal; score 7-10) | 3.6%  N=1308 | 3.8%  N=1228 | 9.2%  N=207 | **0.001** |
| Conduct problems at 16.5  (% abnormal; score 4-10) | 4.4%  N=1306 | 3.2%  N=1226 | 9.7%  N=207 | **<0.001** |
| Locus of control at 16.5  (% external [>median]) | 32.6%  N=1211 | 32.9%  N=1179 | 53.4%  N=178 | **<0.001** |
| Sensation seeking at 17  (mean (SD)) | 51.4 (7.3)  N=976 | 52.7 (7.1)  N=890 | 53.3 (7.2)  N=128 | **<0.001** |
| Stressful life events age 16  (mean (SD)) | 2.8 (2.0)  N=1287 | 2.9 (2.0)  N=1243 | 3.0 (2.4)  N=241 | 0.24 |
| Not in education or employment age 17  (% no) | 9.3%  N=1424 | 10.6%  N=1384 | 20.9%  N=277 | **<0.001** |
| Depression at 17  (ICD-10 diagnosis = % yes) | 7.5%  N=1481 | 7.0%  N=1459 | 9.5%  N=284 | 0.33 |
| Smoking cigarettes at 16.5  (% ≥ weekly) | 7.1%  N=1288 | 10.7%  N=1243 | 20.5%  N=195 | **<0.001** |
| Alcohol use at 16.5  (% weekly) | 12.6%  N=1187 | 15.6%  N=1189 | 19.6%  N=184 | **0.002** |

^*^ P-values stem from Chi-square tests or ANOVAs.

**Supplementary table 3**. **Univariable results on the associations between child antecedents and gambling activity at age 20.**

|  | **Age 20** | | | |
| --- | --- | --- | --- | --- |
| **Variables** | **No gambling**  **(N tot=1259)** | **< Weekly gambling (occasional)**  **(N tot=2200)** | **≥ Weekly gambling (regular)**  **(N tot=481)** | **P-value^*^** |
| Gender (% male) | 31.9% | 39.5% | 59.5% | **<0.001** |
| IQ at 8 (% bottom quartile) | 13.3%  N=999 | 16.6%  N=1740 | 22.5%  N=356 | **<0.001** |
| Plays video games with other children at 13/14  (% yes) | 53.2%  N=965 | 59.6%  N=1624 | 71.1%  N=329 | **<0.001** |
| Hyperactivity at 16.5  (% abnormal; score 7-10) | 3.4%  N=994 | 4.2%  N=1624 | 5.9%  N=337 | 0.13 |
| Conduct problems at 16.5  (% abnormal; score 4-10) | 4.0%  N=992 | 3.8%  N=1624 | 4.2%  N=336 | 0.94 |
| Locus of control at 16.5  (% external [>median]) | 33.9%  N=975 | 36.3%  N=1553 | 44.2%  N=317 | **0.004** |
| Sensation seeking at 17  (mean (SD)) | 51.1 (7.3)  N=808 | 52.4 (7.3)  N=1219 | 52.9 (7.0)  N=231 | **<0.001** |
| Stressful life events age 16  (mean (SD)) | 2.9 (2.0)  N=1036 | 3.0 (2.1)  N=1655 | 2.7 (2.0)  N=331 | 0.19 |
| Not in employment or education/training at 21  (% no) | 8.7%  N=1209 | 7.0%  N=2115 | 11.9%  N=454 | **0.002** |
| Depression at 17  (ICD-10 diagnosis = % yes) | 8.8%  N=865 | 6.4%  N=1419 | 8.1%  N=272 | 0.10 |
| Smoking cigarettes at 21  (% weekly) | 13.4%  N=1228 | 21.1%  N=2158 | 26.2%  N=465 | **<0.001** |
| Alcohol use at 21  (% harmful use) | 8.1%  N=1119 | 13.6%  N=2057 | 18.0%  N=445 | **<0.001** |

^*^ P-values stem from Chi-square tests or ANOVAs.

**Supplementary table 4**. **Univariable results on the associations between child antecedents and gambling activity at age 24.**

|  | **Age 24** | | | |
| --- | --- | --- | --- | --- |
| **Variables** | **No gambling**  **(N tot=1292)** | **< Weekly gambling (occasional)**  **(N tot=2120)** | **≥ Weekly gambling (regular)**  **(N tot=429)** | **P-value^*^** |
| Gender (% male) | 30.0% | 34.9% | 54.6% | **<0.001** |
| IQ at 8 (% bottom quartile) | 16.8%  N=1022 | 16.6%  N=1643 | 21.1%  N=323 | 0.15 |
| Plays video games with other children at 13/14  (% yes) | 52.7%  N=972 | 57.2%  N=1519 | 66.2%  N=305 | **<0.001** |
| Hyperactivity at 16.5  (% abnormal; score 7-10) | 3.2%  N=970 | 3.8%  N=1546 | 5.2%  N=269 | 0.30 |
| Conduct problems at 16.5  (% abnormal; score 4-10) | 4.2%  N=971 | 3.9%  N=1548 | 4.5%  N=268 | 0.89 |
| Locus of control at 16.5  (% external [>median]) | 34.0%  N=950 | 36.8%  N=1436 | 39.5%  N=253 | 0.18 |
| Sensation seeking at 17  (mean (SD)) | 51.2 (7.5)  N=791 | 52.0 (7.3)  N=1147 | 51.8 (7.6)  N=187 | 0.09 |
| Stressful life events age 16  (mean (SD)) | 2.9 (2.0)  N=1009 | 3.0 (2.1)  N=1532 | 2.7 (1.9)  N=267 | 0.23 |
| Not in employment or education/training at 21  (% no) | 6.6%  N=945 | 6.2%  N=1411 | 10.5%  N=257 | **0.04** |
| Diagnosed depression at 23  (% yes) | 21.5%  N=993 | 18.9%  N=1474 | 19.3%  N=275 | 0.28 |
| Smoking cigarettes at 23  (% weekly) | 10.0%  N=998 | 14.7%  N=1490 | 22.6%  N=275 | **<0.001** |
| Alcohol abuse at 23 (DSM4)  (% yes) | 6.7%  N=987 | 9.6%  N=1475 | 11.6%  N=267 | **0.01** |
| Social media use at 24  (% >10 times a day) | 33.7%  N=1256 | 40.4%  N=2078 | 43.7%  N=414 | **<0.001** |

^*^ P-values stem from Chi-square tests or ANOVAs.

**Supplementary table 5**. **Univariable results on the associations between parental and socioeconomic antecedents and gambling activity at age 17.**

|  | **Age 17** | | | |
| --- | --- | --- | --- | --- |
| **Variables** | **No gambling**  **(N tot=1632)** | **< Weekly gambling (occasional)**  **(N tot=1612)** | **≥ Weekly gambling (regular)**  **(N tot=322)** | **P-value^*^** |
| Maternal age at birth  (% < mean) | 28.1%  N=1564 | 34.8%  N=1528 | 44.0%  N=300 | **<0.001** |
| Maternal education pregnancy  (% with degree higher than A level) | 26.1%  N=1595 | 18.2%  N=1567 | 7.8%  N=309 | **<0.001** |
| Crowding index in pregnancy  (% >1) | 3.2%  N=1578 | 3.6%  N=1549 | 5.8%  N=311 | **<0.001** |
| Financial difficulties in pregnancy  (% difficulty score >3) | 23.2%  N=1555 | 24.8%  N=1525 | 26.8%  N=299 | **0.03** |
| Maternal depression (child age 10-12)  (% yes) | 19.8%  N=1329 | 19.7%  N=1302 | 22.2%  N=234 | 0.67 |
| Partner’s depression (child age 10-12)  (% yes) | 19.6%  N=1124 | 18.6%  N=1082 | 14.4%  N=180 | 0.26 |
| Index of Multiple Deprivation (child age 11)  (% most deprived quintile) | 17.0%  N=1372 | 15.9%  N=1314 | 20.2%  N=247 | 0.35 |
| Housing child age 18  (% Council/housing association) | 2.3%  N=1120 | 4.4%  N=1018 | 8.1%  N=161 | **0.002** |
| Maternal gambling child age 6  (% ≥ Weekly) | 32.1%  N=1448 | 42.2%  N=1376 | 53.8%  N=253 | **<0.001** |
| Paternal gambling child age 6  (% ≥ Weekly) | 38.2%  N=921 | 53.4%  N=835 | 67.1%  N=140 | **<0.001** |
| Mother’s gambling child age 18  (% Low-High risk gambler) | 1.7%  N=1110 | 1.9%  N=1014 | 3.8%  N=159 | **<0.001** |

^*^ P-values stem from Chi-square tests, ANOVAs or Kruskal-Wallis tests.

**Supplementary table 6**. **Univariable results on the associations between parental and socioeconomic antecedents and gambling activity at age 20.**

|  | **Age 20** | | | |
| --- | --- | --- | --- | --- |
| **Variables** | **No gambling**  **(N tot=1259)** | **< Weekly gambling**  **(N tot=2200)** | **≥ Weekly gambling (N tot=481)** | **P-value^*^** |
| Maternal age at birth  (% < mean) | 29.7%  N=1206 | 34.7%  N=2088 | 41.9%  N=442 | **<0.001** |
| Maternal education pregnancy  (% with degree higher than A level) | 29.4%  N=1237 | 16.7%  N=2137 | 9.6%  N=467 | **<0.001** |
| Crowding index in pregnancy  (% >1) | 2.9%  N=1223 | 3.4%  N=2117 | 3.9%  N=458 | **<0.001** |
| Financial difficulties in pregnancy  (% difficulty score >3) | 21.8%  N=1207 | 25.9%  N=2088 | 30.1%  N=455 | **0.001** |
| Maternal depression (child age 10-12)  (% yes) | 19.7%  N=1018 | 20.5%  N=1755 | 23.0%  N=357 | 0.41 |
| Partner’s depression (child age 10-12)  (% yes) | 18.6%  N=871 | 17.9%  N=1445 | 14.7%  N=292 | 0.32 |
| Index of Multiple Deprivation (child age 11)  (% most deprived quintile) | 14.9%  N=1038 | 16.5%  N=1786 | 22.1%  N=357 | 0.08 |
| Housing child age 18  (% Council/housing association) | 2.9%  N=853 | 3.7%  N=1339 | 9.0%  N=266 | **<0.001** |
| Maternal gambling child age 6  (% ≥ Weekly) | 28.9%  N=1123 | 42.6%  N=1902 | 47.9%  N=399 | **<0.001** |
| Paternal gambling child age 6  (% ≥ Weekly) | 35.2%  N=718 | 53.2%  N=1148 | 58.8%  N=211 | **<0.001** |
| Mother’s gambling child age 18  (% Low-High risk gambler) | 1.3%  N=848 | 2.0%  N=1330 | 5.0%  N=261 | **<0.001** |

^*^ P-values stem from Chi-square tests, ANOVAs or Kruskal-Wallis tests.

**Supplementary table 7**. **Univariable results on the associations between parental and socioeconomic antecedents and gambling activity at age 24.**

|  | **Age 24** | | | |
| --- | --- | --- | --- | --- |
| **Variables^*^** | **No gambling**  **(N tot=1292)** | **< Weekly gambling**  **(N tot=2120)** | **≥ Weekly gambling (N tot=429)** | **P-value^**^** |
| Maternal age at birth  (% < mean) | 28.8%  N=1219 | 36.2%  N=1992 | 42.3%  N=400 | **<0.001** |
| Maternal education in pregnancy  (% with degree higher than A level) | 27.4%  N=1251 | 17.3%  N=2044 | 10.5%  N=408 | **<0.001** |
| Crowding index pregnancy  (% >1) | 3.6%  N=1248 | 3.5%  N=2024 | 4.6%  N=412 | **0.003** |
| Financial difficulties in pregnancy  (% difficulty score >3) | 23.2%  N=1218 | 24.4%  N=1997 | 30.7%  N=401 | **0.007** |
| Maternal depression (child age 10-12)  (% yes) | 20.1%  N=1005 | 20.8%  N=1626 | 20.2%  N=307 | 0.91 |
| Partner’s depression (child age 10-12)  (% yes) | 20.4%  N=845 | 17.4%  N=1331 | 17.3%  N=249 | 0.19 |
| Index of Multiple Deprivation (child age 11)  (% most deprived quintile) | 17.1%  N=1031 | 15.8%  N=1651 | 20.8%  N=312 | **0.002** |
| Housing child age 18  (% Council/housing association) | 3.5%  N=806 | 3.4%  N=1227 | 9.6%  N=229 | **<0.001** |
| Maternal gambling child age 6  (% ≥ Weekly) | 29.0%  N=1124 | 40.4%  N=1794 | 55.5%  N=355 | **<0.001** |
| Paternal gambling child age 6  (% ≥ Weekly) | 38.3%  N=711 | 50.8%  N=1075 | 59.6%  N=203 | **<0.001** |
| Mother’s gambling child age 18  (% Low-High risk gambler) | 1.6%  N=800 | 2.0%  N=1220 | 5.2%  N=229 | **<0.001** |

**^*^** Variables are explained in supplementary material 1. ^**^ P-values stem from Chi-square tests, ANOVAs or Kruskal-Wallis tests.

**Supplementary table 8**. **Unadjusted and adjusted multinomial odds ratios (OR) and 95% confidence intervals (CI) for *child variables only* at age 17**. Imputed data stratified by gender.

|  | **Males (N=2486)** | | | | **Females (N=3495)** | | | |
| --- | --- | --- | --- | --- | --- | --- | --- | --- |
|  | **No gambling vs. < weekly gambling** | | **No gambling vs. Weekly gambling** | | **No gambling vs. < weekly gambling** | | **No gambling vs. Weekly gambling** | |
| **Variables** | **Unadjusted OR (95% CI)** | **Adjusted OR (95% CI)** | **Unadjusted OR (95% CI)** | **Adjusted OR (95% CI)** | **Unadjusted OR (95% CI)** | **Adjusted OR (95% CI)** | **Unadjusted OR (95% CI)** | **Adjusted OR (95% CI)** |
| IQ at 8  - bottom quartile | 1.06 (0.76, 1.47) | 1.17 (0.84, 1.63) | **2.58 (1.78, 3.73)** | **2.37 (1.56, 3.59)** | 0.99 (0.80, 1.24) | 1.02 (0.81, 1.27) | **2.31 (1.50, 3.56)** | **1.74 (1.09, 2.79)** |
| Playing video games with friends at 13/14 | 1.12 (0.83, 1.52) | 1.17 (0.86, 1.58) | 1.05 (0.70, 1.55) | 1.19 (0.78, 1.81) | 1.10 (0.92, 1.32) | 1.12 (0.93, 1.35) | 1.11 (0.75, 1.63) | 1.15 (0.77, 1.71) |
| Hyperactivity at 16.5  - abnormal; score 7-10 | 1.11 (0.66, 1.85) | 1.10 (0.63, 1.90) | **2.08 (1.08, 4.03)** | 1.32 (0.62, 2.83) | 1.06 (0.63, 1.81) | 1.06 (0.60, 1.88) | **2.75 (1.35, 5.63)** | 1.56 (0.70, 3.50) |
| Conduct problems at 16.5  - abnormal; score 4-10 | 0.71 (0.38, 1.34) | 0.63 (0.32, 1.25) | **2.45 (1.30, 4.64)** | 1.42 (0.68, 2.96) | 0.83 (0.51, 1.33) | 0.72 (0.44, 1.20) | **2.92 (1.52, 5.64)** | 1.39 (0.67, 2.91) |
| Locus of control at 16.5  - >median [external] | 0.98 (0.76, 1.27) | 0.96 (0.74, 1.25) | **2.68 (1.92, 3.74)** | **2.22 (1.57, 3.15)** | 1.07 (0.87, 1.31) | 1.03 (0.84, 1.27) | **2.86 (1.92, 4.26)** | **2.10 (1.37, 3.20)** |
| Sensation seeking at 17 | **1.03 (1.01, 1.05)** | **1.02 (1.00, 1.05)** | 1.03 (1.00, 1.05) | 1.02 (0.99, 1.05) | **1.02 (1.00, 1.03**) | 1.01 (0.99, 1.03) | 1.01 (0.98, 1.04) | 1.00 (0.97, 1.04) |
| In education or employment age 17 - no | 1.10 (0.76, 1.59) | 1.06 (0.72, 1.56) | **2.17 (1.39, 3.39)** | 1.54 (0.92, 2.59) | 1.14 (0.83, 1.56) | 1.08 (0.78, 1.50) | **2.91 (1.83, 4.63)** | **1.86 (1.13, 3.07)** |
| Smoking cigarettes at 16.5  - tried  - <weekly  - ≥ weekly | **1.42 (1.07, 1.88)**  **1.89 (1.11, 3.21)**  1.45 (0.94, 2.23) | 1.30 (0.97, 1.75)  1.67 (0.96, 2.90)  1.28 (0.79, 2.08) | **1.89 (1.25, 2.85)**  **2.27 (1.10, 4.70)**  **3.16 (1.87, 5.34)** | **1.61 (1.02, 2.54)**  1.72 (0.80, 3.70)  **2.10 (1.13, 3.93)** | 1.23 (0.99, 1.54)  1.34 (0.95, 1.89)  **1.68 (1.27, 2.23)** | 1.19 (0.94, 1.50)  1.27 (0.89, 1.81)  **1.60 (1.16, 2.21)** | **1.67 (1.05, 2.66)**  1.72 (0.79, 3.71)  **4.66 (2.86, 7.59)** | 1.53 (0.93, 2.50)  1.64 (0.72, 3.73)  **3.45 (1.92, 6.21)** |
| Alcohol use at 16.5  - >once per month/< weekly  - ≥ weekly | **1.40 (1.09, 1.80)**  **1.48 (1.05, 2.09)** | 1.26 (0.98, 1.64)  1.24 (0.85, 1.80) | **1.61 (1.07, 2.42)**  **1.94 (1.18, 3.22)** | 1.49 (0.95, 2.34)  1.46 (0.82, 2.61) | 1.17 (0.95, 1.45)  1.35 (0.99, 1.84) | 1.07 (0.86, 1.34)  1.09 (0.77, 1.55) | 1.11 (0.73, 1.67)  1.50 (0.84, 2.67) | 0.93 (0.58, 1.49)  0.89 (0.45, 1.73) |

**Supplementary table 9**. **Unadjusted and adjusted multinomial odds ratios (OR) and 95% confidence intervals (CI) for *child variables only* at age 20**. Imputed data stratified by gender.

|  | **Males (N=2486)** | | | | **Females (N=3495)** | | | |
| --- | --- | --- | --- | --- | --- | --- | --- | --- |
|  | **No gambling vs. < weekly gambling** | | **No gambling vs. Weekly gambling** | | **No gambling vs. < weekly gambling** | | **No gambling vs. Weekly gambling** | |
| **Variables** | **Unadjusted OR (95% CI)** | **Adjusted OR (95% CI)** | **Unadjusted OR (95% CI)** | **Adjusted OR (95% CI)** | **Unadjusted OR (95% CI)** | **Adjusted OR (95% CI)** | **Unadjusted OR (95% CI)** | **Adjusted OR (95% CI)** |
| IQ at 8  - bottom quartile | 1.26 (0.90, 1.75) | **1.60 (1.13, 2.28)** | **1.94 (1.30, 2.90)** | **2.30 (1.49, 3.57)** | **1.32 (1.04, 1.68)** | **1.43 (1.10, 1.85)** | **1.97 (1.30, 3.00)** | **1.85 (1.18, 2.91)** |
| Playing video games with friends at 13/14 | 1.04 (0.76, 1.42) | 1.13 (0.82, 1.56) | 1.20 (0.80, 1.82) | 1.37 (0.90, 2.09) | 1.20 (1.00, 1.43) | **1.22 (1.02, 1.48)** | 1.42 (0.98, 2.04) | **1.46 (1.01, 2.13)** |
| Hyperactivity at 16.5  - abnormal; score 7-10 | 0.90 (0.53, 1.52) | 0.85 (0.48, 1.50) | 1.46 (0.84, 2.52) | 1.20 (0.64, 2.25) | 1.24 (0.72, 2.16) | 1.18 (0.65, 2.13) | 1.82 (0.84, 3.94) | 1.31 (0.57, 3.02) |
| Conduct problems at 16.5  - abnormal; score 4-10 | 0.92 (0.51, 1.64) | 0.83 (0.44, 1.57) | 1.12 (0.51, 2.45) | 0.71 (0.29, 1.74) | 0.95 (0.62, 1.44) | 0.75 (0.48, 1.19) | 1.24 (0.54, 2.81) | 0.69 (0.28, 1.67) |
| Locus of control at 16.5  - >median [external] | 1.03 (0.79, 1.35) | 0.98 (0.74, 1.30) | **1.56 (1.14, 2.14)** | 1.36 (0.97, 1.91) | **1.22 (1.01, 1.48)** | 1.16 (0.95, 1.42) | **1.87 (1.34, 2.62)** | **1.58 (1.12, 2.24)** |
| Sensation seeking at 17 | **1.03 (1.02, 1.06)** | **1.02 (1.00, 1.04)** | **1.04 (1.01, 1.06)** | 1.02 (0.99, 1.05) | **1.02 (1.00, 1.03)** | 1.01 (1.00, 1.03) | 1.02 (0.99, 1.04) | 1.01 (0.98, 1.04) |
| In education or employment age 21 - no | 0.78 (0.53, 1.15) | 0.81 (0.53, 1.24) | 1.40 (0.85, 2.30) | 1.33 (0.77, 2.29) | 0.82 (0.60, 1.11) | 0.75 (0.54, 1.04) | 1.54 (0.93, 2.53) | 1.17 (0.70, 1.95) |
| Smoking cigarettes at 21  - ≥ weekly | **1.96 (1.37, 2.80)** | **1.66 (1.13, 2.42)** | **2.58 (1.73, 3.83)** | **1.93 (1.26, 2.95)** | **1.75 (1.38, 2.21)** | **1.62 (1.26, 2.09)** | **2.71 (1.89, 3.88)** | **2.40 (1.63, 3.53)** |
| Alcohol use at 21  - hazardous  - harmful | **1.93 (1.50, 2.50)**  **3.07 (1.98, 4.77)** | **1.81 (1.40, 2.35)**  **2.70 (1.73, 4.20)** | **2.12 (1.54, 2.91)**  **4.45 (2.74, 7.22)** | **2.14 (1.53, 2.98)**  **4.31 (2.59, 7.18)** | **1.45 (1.21, 1.73)**  **1.80 (1.34, 2.43)** | **1.40 (1.16, 1.69)**  **1.60 (1.17, 2.19)** | 1.24 (0.88, 1.75)  1.53 (0.90, 2.59) | 1.25 (0.88, 1.77)  1.32 (0.75, 2.31) |

.

**Supplementary table 10. Unadjusted and adjusted multinomial odds ratios (OR) and 95% confidence intervals (CI) for *child variables only* at age 24.** Imputed data stratified by gender.

|  | **Males (N=2486)** | | | | **Females (N=3495)** | | | |
| --- | --- | --- | --- | --- | --- | --- | --- | --- |
|  | **No gambling vs. < weekly gambling** | | **No gambling vs. Weekly gambling** | | **No gambling vs. < weekly gambling** | | **No gambling vs. Weekly gambling** | |
| **Variables** | **Unadjusted OR (95% CI)** | **Adjusted OR (95% CI)** | **Unadjusted OR (95% CI)** | **Adjusted OR (95% CI)** | **Unadjusted OR (95% CI)** | **Adjusted OR (95% CI)** | **Unadjusted OR (95% CI)** | **Adjusted OR (95% CI)** |
| IQ at 8  - bottom quartile | 0.98 (0.70, 1.36) | 1.02 (0.72, 1.44) | 1.35 (0.90, 2.04) | 1.27 (0.81, 1.98) | 1.11 (0.89, 1.37) | 1.09 (0.87, 1.37) | **1.64 (1.10, 2.44)** | 1.35 (0.88, 2.07) |
| Playing video games with friends at 13/14 | 0.99 (0.73, 1.35) | 1.01 (0.73, 1.39) | 0.99 (0.67, 1.46) | 1.03 (0.69, 1.54) | 1.13 (0.93, 1.38) | 1.11 (0.91, 1.35) | 1.00 (0.69, 1.44) | 1.00 (0.69, 1.47) |
| Hyperactivity at 16.5  - abnormal; score 7-10 | 1.25 (0.72, 2.18) | 1.31 (0.73, 2.36) | 1.50 (0.72, 3.16) | 1.51 (0.69, 3.30) | 1.08 (0.59, 1.99) | 1.07 (0.56, 2.04) | 1.80 (0.80, 4.05) | 1.46 (0.61, 3.50) |
| Conduct problems at 16.5  - abnormal; score 4-10 | 0.90 (0.50, 1.64) | 0.70 (0.37, 1.34) | 0.94 (0.43, 2.07) | 0.56 (0.25, 1.28) | 0.99 (0.62, 1.59) | 0.85 (0.50, 1.42) | 1.24 (0.53, 2.88) | 0.64 (0.26, 1.59) |
| Locus of control at 16.5  - >median [external] | 1.12 (0.86, 1.47) | 1.09 (0.82, 1.45) | **1.44 (1.02, 2.02)** | 1.32 (0.93, 1.87) | 1.14 (0.95, 1.38) | 1.11 (0.91, 1.35) | **1.62 (1.14, 2.29)** | **1.43 (1.00, 2.06)** |
| Sensation seeking at 17 | **1.02 (1.00, 1.04)** | 1.01 (0.99, 1.03) | 1.01 (0.98, 1.03) | 1.00 (0.97, 1.02) | 1.01 (0.99, 1.02) | 1.00 (0.99, 1.02) | 0.99 (0.96, 1.01) | 0.98 (0.96, 1.01) |
| In education or employment age 21  - no | 0.90 (0.57, 1.43) | 0.87 (0.54, 1.42) | 1.27 (0.71, 2.30) | 1.12 (0.61, 2.07) | 1.04 (0.73, 1.47) | 0.99 (0.69, 1.43) | **1.74 (1.03, 2.97)** | 1.35 (0.76, 2.39) |
| Smoking cigarettes at 23  - ≥ weekly | **1.82 (1.25, 2.66)** | **1.74 (1.17, 2.60)** | **2.43 (1.57, 3.78)** | **2.38 (1.49, 3.79)** | **1.39 (1.08, 1.78)** | **1.39 (1.06, 1.83)** | **3.02 (1.97, 4.63)** | **3.00 (1.88, 4.79)** |
| Alcohol abuse at 23  - yes | **1.60 (1.01, 2.53)** | 1.39 (0.86, 2.24) | **1.84 (1.07, 3.16)** | 1.52 (0.84, 2.75) | 1.32 (0.91, 1.91) | 1.21 (0.83, 1.77) | 1.35 (0.74, 2.47) | 1.09 (0.56, 2.11) |
| Social media use at 24  -2-10 times/day  - >10 times/day | **1.56 (1.10, 2.19)**  **1.92 (1.32, 2.78)** | **1.56 (1.10, 2.21)**  **1.92 (1.31, 2.83)** | **1.65 (1.01, 2.67)**  **2.66 (1.60, 4.40)** | **1.69 (1.03, 2.75)**  **2.74 (1.63, 4.61)** | 1.36 (0.98, 1.88)  **1.81 (1.31, 2.51)** | 1.36 (0.98, 1.89)  **1.80 (1.29, 2.50)** | 1.85 (0.89, 3.83)  **2.69 (1.28, 5.63)** | 2.08 (0.97, 4.45)  **2.97 (1.37, 6.45)** |

**Supplementary table 11. Adjusted multinomial odds ratios for parental and socioeconomic antecedents on *occasional* gambling at age 17, 20 and 24**. Imputed data set (N=5981).

|  | ***Age 17*** | | |
| --- | --- | --- | --- |
| **Males** | **Model 1**  **OR (95% CI)^*^** | **Model 2**  **OR (95% CI)^*^** | **Model 3**  **OR (95% CI)^*^** |
| IQ at 8  - bottom quartile | 1.17 (0.84, 1.63)^**^ | 1.08 (0.76, 1.53) | 1.09 (0.76, 1.57) |
| Locus of control at 16.5  - >median [external] | 0.96 (0.74, 1.25) ^**^ | 0.90 (0.68, 1.18) | 0.89 (0.68, 1.18) |
| Sensation seeking at 17 | **1.02 (1.00, 1.05)** | **1.03 (1.01, 1.05)** | **1.03 (1.01, 1.05)** |
| Smoking cigarettes at 16.5  - tried  - <weekly  - ≥ weekly | 1.30 (0.97, 1.75) ^**^  1.67 (0.96, 2.90)  1.28 (0.79, 2.08) | **1.37 (1.02, 1.84)**  **1.73 (1.06, 3.00)**  1.28 (0.80, 2.05) | **1.36 (1.01, 1.84)**  1.73 (1.00, 3.00)  1.28 (0.80, 2.06) |
|  | *Age 17* | | |
| **Females** | **Model 1**  **OR (95% CI)^*^** | **Model 2**  **OR (95% CI)^*^** | **Model 3**  **OR (95% CI)^*^** |
| IQ at 8  - bottom quartile | 1.02 (0.81, 1.27) ^**^ | 0.90 (0.71, 1.13) | 0.89 (0.70, 1.13) |
| Locus of control at 16.5  - >median [external] | 1.03 (0.84, 1.27) ^**^ | 0.97 (0.78, 1.20) | 0.96 (0.77, 1.20) |
| Smoking cigarettes at 16.5  - tried  - <weekly  - ≥ weekly | 1.19 (0.94, 1.50)  1.27 (0.89, 1.81)  **1.60 (1.16, 2.21)** | 1.25 (0.99, 1.57)  1.34 (0.94, 1.91)  **1.63 (1.21, 2.19)** | 1.25 (0.99, 1.58)  1.35 (0.95, 1.93)  **1.62 (1.21, 2.18)** |
|  | ***Age 20*** | | |
| **Males** | **Model 1**  **OR (95% CI)^*^** | **Model 2**  **OR (95% CI)^*^** | **Model 3**  **OR (95% CI)^*^** |
| IQ at 8  - bottom quartile | **1.60 (1.13, 2.28)** | 1.33 (0.93, 1.91) | 1.34 (092, 1.94) |
| Sensation seeking at 17 | **1.02 (1.00, 1.04)** | **1.03 (1.01, 1.05)** | **1.03 (1.01, 1.05)** |
| Smoking cigarettes at 21  - ≥ weekly | **1.66 (1.13, 2.42)** | **1.48 (1.02, 2.15)** | **1.48 (1.03, 2.14)** |
| Alcohol use at 21  - hazardous  - harmful | **1.81 (1.40, 2.35)**  **2.70 (1.73, 4.20)** | **1.94 (1.48, 2.55)**  **3.05 (1.92, 4.83)** | **1.94 (1.47, 2.55)**  **3.03 (1.90, 4.83)** |
|  | ***Age 20*** | | |
| **Females** | **Model 1**  **OR (95% CI)^*^** | **Model 2**  **OR (95% CI)^*^** | **Model 3**  **OR (95% CI)^*^** |
| IQ at 8  - bottom quartile | **1.43 (1.10, 1.85)** | 1.09 (0.82, 1.44) | 1.06 (0.80, 1.41) |
| Playing video games with friends at 13/14 | **1.22 (1.02, 1.48)** | 1.21 (1.00, 1.47) | 1.22 (1.00, 1.48) |
| Locus of control at 16.5  - >median [external] | 1.16 (0.95, 1.42) ^**^ | 0.98 (0.80, 1.20) | 0.97 (0.78, 1.19) |
| Smoking cigarettes at 21  - ≥ weekly | **1.62 (1.26, 2.09)** | **1.52 (1.17, 1.97)** | **1.51 (1.16, 1.97)** |
| Alcohol use at 21  - hazardous  - harmful | **1.40 (1.16, 1.69)**  **1.60 (1.17, 2.19)** | **1.55 (1.27, 1.88)**  **1.87 (1.37, 2.57)** | **1.57 (1.28, 1.92)**  **1.91 (1.39, 2.64)** |
|  | ***Age 24*** | | |
| **Males** | **Model 1**  **OR (95% CI)^*^** | **Model 2**  **OR (95% CI)^*^** | **Model 3**  **OR (95% CI)^*^** |
| Smoking cigarettes at 23  - ≥ weekly | **1.74 (1.17, 2.60)** | **1.76 (1.21, 2.58)** | **1.84 (1.26, 2.70)** |
| Social media use at 24  -2-10 times/day  - >10 times/day | **1.56 (1.10, 2.21)**  **1.92 (1.31, 2.83)** | **1.60 (1.13, 2.29)**  **2.01 (1.36, 2.98)** | **1.63 (1.13, 2.33)**  **2.04 (1.37, 3.05)** |
|  | ***Age 24*** | | |
| **Females** | **Model 1**  **OR (95% CI)^*^** | **Model 2**  **OR (95% CI)^*^** | **Model 3**  **OR (95% CI)^*^** |
| Locus of control at 16.5  - >median [external] | 1.11 (0.91, 1.35) ^**^ | 0.96 (0.78, 1.18) | 0.96 (0.78, 1.18) |
| Smoking cigarettes at 23  - ≥ weekly | **1.39 (1.06, 1.83)** | **1.31 (1.00, 1.72)** | **1.34 (1.02, 1.77)** |
| Social media use at 24  -2-10 times/day  - >10 times/day | 1.36 (0.98, 1.89)  **1.80 (1.29, 2.50)** | 1.30 (0.93, 1.82)  **1.72 (1.22, 2.42)** | 1.30 (0.93, 1.81)  **1.71 (1.22, 2.41)** |

^*^ Model 1: adjusted for other child variables, Model 2: model 1 + adjusted for maternal age, maternal education, maternal gambling at 6 and 18 years, paternal gambling at 6 years, model 3: model 2+ adjusted for crowding index, financial difficulties, housing status and Index of Multiple Deprivation.

^**^ These variables were kept in as regular gambling was significantly associated with them (shown in main text table 4).

**Supplementary table 12. Individual effects of parental and socioeconomic antecedents on *occasional and regular gambling* at age 17, 20 and 24.**

Fully adjusted (child, parental and socioeconomic) multinomial odds ratios and 95% confidence intervals on imputed data set (N=5981).

| **Males only** | **Age 17** | | **Age 20** | | **Age 24** | |
| --- | --- | --- | --- | --- | --- | --- |
| ***Fully adjusted model*** | **< Weekly gambling (occasional)** | **≥ Weekly gambling**  **(regular)** | **< Weekly gambling (occasional)** | **≥ Weekly gambling**  **(regular)** | **< Weekly gambling (occasional)** | **≥ Weekly gambling**  **(regular)** |
| Maternal age at birth  - < mean | 1.09 (0.84, 1.41) | 1.10 (0.77, 1.57) | 1.04 (0.79, 1.37) | 1.16 (0.81, 1.64) | 1.28 (0.97, 1.70) | 1.36 (0.95, 1.95) |
| Maternal education pregnancy  - degree higher than A level | 0.82 (0.51, 1.31) | **0.27 (0.14, 0.53)** | 0.74 (0.44, 1.25) | **0.34 (0.17, 0.68)** | **0.58 (0.35, 0.96)** | **0.38 (0.20, 0.74)** |
| Maternal gambling child age 6  - <weekly  - ≥ weekly | 1.18 (0.85, 1.64)  1.13 (0.81, 1.57) | 1.36 (0.82, 2.25)  1.53 (0.90, 2.62) | **1.56 (1.12, 2.19)**  **1.58 (1.14, 2.20)** | **1.88 (1.20, 2.93)**  **2.06 (1.31, 3.26)** | **1.55 (1.09, 2.20)**  1.32 (0.95, 1.83) | **1.78 (1.11, 2.86)**  **2.58 (1.70, 3.91)** |
| Paternal gambling child age 6  - < weekly  - ≥ weekly | 1.39 (1.00, 2.03)  **1.75 (1.25, 2.46)** | 1.53 (0.84, 2.81)  **2.19 (1.12, 4.29)** | 1.37 (0.94, 1.99)  **1.67 (1.12, 2.50)** | **1.67 (1.00, 2.78)**  **2.23 (1.25, 3.98)** | 1.38 (0.98, 1.96)  **1.52 (1.05, 2.19)** | 1.59 (0.95, 2.66)  **1.84 (1.09, 3.10)** |
| Mother’s gambling child age 18  - no problem gambler  - low-high risk gambler | **1.55 (1.18, 2.03)**  1.07 (0.48, 2.38) | **1.51 (1.01, 2.26)**  1.74 (0.60, 5.10) | 1.40 (0.99, 1.99)  1.09 (0.44, 2.74) | 1.34 (0.84, 2.15)  1.43 (0.49, 4.15) | 1.37 (0.99, 1.88)  1.28 (0.42, 3.91) | 1.27 (0.84, 1.92)  1.61 (0.53, 4.88) |
| Crowding index pregnancy  - >1 | 0.63 (0.34, 1.17) | 0.70 (0.26, 1.87) | 0.71 (0.34, 1.48) | 0.61 (0.23, 1.60) | 0.56 (0.28, 1.13) | 0.39 (0.14, 1.05) |
| Financial difficulties pregnancy  - difficulty score >3 | 0.88 (0.65, 1.19) | 0.66 (0.42, 1.03) | 1.08 (0.78, 1.50) | 0.89 (0.59, 1.34) | **0.74 (0.54, 1.00)** | 0.88 (0.56, 1.36) |
| Index of Multiple Deprivation (child age 11)  - 5^th^ most deprived quintile | 0.81 (0.53, 1.21) | 0.76 (0.42, 1.37) | 1.02 (0.65, 1.62) | 1.03 (0.59, 1.83) | 0.93 (0.61, 1.41) | 1.04 (0.57, 1.87) |
| Housing child age 18  - council/housing association | 1.45 (0.70, 3.02) | 1.77 (0.67, 4.69) | 0.86 (0.40, 1.82) | 1.51 (0.67, 3.41) | 1.02 (0.47, 2.22) | - 1. (0.88, 4.59) |
|  | | | | | | |
| **Females only** | **Age 17** | | **Age 20** | | **Age 24** | |
|  | **< Weekly gambling (occasional)** | **≥ Weekly gambling**  **(regular)** | **< Weekly gambling (occasional)** | **≥ Weekly gambling**  **(regular)** | **< Weekly gambling (occasional)** | **≥ Weekly gambling**  **(regular)** |
| Maternal age at birth  - < mean | **1.23 (1.01, 1.50)** | 1.29 (0.86, 1.94) | 1.03 (0.83, 1.27) | 1.19 (0.84, 1.70) | 1.13 (0.94, 1.37) | 1.33 (0.95, 1.87) |
| Maternal education pregnancy  - degree higher than A level | 0.89 (0.62, 1.27) | **0.15 (0.05, 0.47)** | **0.47 (0.32, 0.68)** | **0.18 (0.08, 0.40)** | **0.43 (0.30, 0.63)** | **0.37 (0.18, 0.77)** |
| Maternal gambling child age 6  - <weekly  - ≥ weekly | 1.19 (0.92, 1.54)  1.12 (0.85, 1.48) | 1.49 (0.83, 2.68)  1.51 (0.80, 2.85) | **1.44 (1.12, 1.85)**  **1.35 (1.04, 1.74)** | 1.53 (0.96, 2.44)  1.44 (0.86, 2.40) | **1.50 (1.18, 1.90)**  **1.51 (1.17, 1.94)** | 1.51 (0.90, 2.52)  **2.43 (1.50, 3.93)** |
| Paternal gambling child age 6  - < weekly  - ≥ weekly | 1.15 (0.86, 1.54)  **1.64 (1.18, 2.27)** | 1.17 (0.56, 2.42)  1.72 (0.77, 3.82) | 1.32 (1.00, 1.76)  **1.89 (1.39, 2.57)** | 1.33 (0.75, 2.36)  **2.23 (1.20, 4.12)** | 1.21 (0.91, 1.60)  **1.49 (1.11, 2.00)** | 1.26 (0.74, 2.16)  1.50 (0.86, 2.62) |
| Mother’s gambling child age 18  - no problem gambler  - low-high risk gambler | **1.61 (1.25, 2.07)**  **1.99 (1.02, 3.89)** | 1.50 (0.92, 2.45)  2.52 (0.74, 8.53) | **1.45 (1.14, 1.84)**  1.88 (0.89, 3.98) | 1.34 (0.83, 2.18)  2.48 (0.97, 6.37) | **1.36 (1.07, 1.75)**  1.32 (0.68, 2.57) | 1.33 (0.81, 2.19)  1.69 (0.63, 4.51) |
| Crowding index pregnancy  - >1 | 1.09 (0.64, 1.84) | 1.57 (0.64, 3.83) | 1.06 (0.61, 1.84) | 1.12 (0.49, 2.58) | 0.78 (0.44, 1.38) | 0.79 (0.34, 1.81) |
| Financial difficulties pregnancy  - difficulty score >3 | 0.90 (0.70, 1.18) | 0.96 (0.59, 1.58) | 1.05 (0.82, 1.35) | 1.09 (0.72, 1.64) | 1.00 (0.77, 1.28) | 0.88 (0.57, 1.34) |
| Index of Multiple Deprivation (child age 11)  - 5^th^ most deprived quintile | 0.95 (0.68, 1.33) | 1.13 (0.60, 2.14) | 1.05 (0.76, 1.45) | 1.39 (0.82, 2.36) | 0.91 (0.66, 1.25) | 1.02 (0.57, 1.82) |
| Housing child age 18  - council/housing association | 1.58 (0.88, 2.84) | 1.40 (0.59, 3.35) | 1.04 (0.60, 1.81) | 1.75 (0.81, 3.80) | 1.00 (0.58, 1.71) | **2.60 (1.31, 5.14)** |

**Supplementary table 13. Summary table of fully adjusted multinomial odds ratios for *occasional* gambling in males and females at age 17, 20 and 24**.

Fully adjusted multinomial odds ratios for less than weekly gambling. Only those significant after full adjustment at one or more time points are shown. The highlighted sections are either non-significant or not measured at that age. Imputed data set (N=5981).

|  | **Males** | | | **Females** | | |
| --- | --- | --- | --- | --- | --- | --- |
| **Fully adjusted ORs (95% CI)** | **Age 17** | **Age 20** | **Age 24** | **Age 17** | **Age 20** | **Age 24** |
| Sensation seeking at 17 | **1.03 (1.01, 1.05)** | **1.03 (1.01, 1.05)** |  |  |  |  |
| Smoking cigarettes at 16.5  - tried  - <weekly  - ≥ weekly | **1.36 (1.01, 1.84)**  1.73 (1.00, 3.00)  1.28 (0.80, 2.06) |  |  | 1.25 (0.99, 1.58)  1.35 (0.95, 1.93)  **1.62 (1.21, 2.18)** |  |  |
| Smoking cigarettes at 21  - ≥ weekly |  | **1.48 (1.03, 2.14)** |  |  | **1.51 (1.16, 1.97)** |  |
| Alcohol use at 21  - hazardous  - harmful |  | **1.94 (1.47, 2.55)**  **3.03 (1.90, 4.83)** |  |  | **1.57 (1.28, 1.92)**  **1.91 (1.39, 2.64)** |  |
| Smoking cigarettes at 23  - ≥ weekly |  |  | **1.84 (1.26, 2.70)** |  |  | **1.34 (1.02, 1.77)** |
| Social media use at 24  -2-10 times/day  - >10 times/day |  |  | **1.63 (1.13, 2.33)**  **2.04 (1.37, 3.05)** |  |  | 1.30 (0.93, 1.81)  **1.71 (1.22, 2.41)** |
| Maternal age at birth  - < mean |  |  |  | **1.23 (1.01, 1.50)** |  |  |
| Maternal education pregnancy  - degree higher than A level |  |  | **0.58 (0.35, 0.96)** |  | **0.47 (0.32, 0.68)** | **0.43 (0.30, 0.63)** |
| Maternal gambling child age 6  - <weekly  - ≥ weekly |  | **1.56 (1.12, 2.19)**  **1.58 (1.14, 2.20)** | **1.55 (1.09, 2.20)**  1.32 (0.95, 1.83) |  | **1.44 (1.12, 1.85)**  **1.35 (1.04, 1.74)** | **1.50 (1.18, 1.90)**  **1.51 (1.17, 1.94)** |
| Paternal gambling child age 6  - < weekly  - ≥ weekly | 1.39 (1.00, 2.03)  **1.75 (1.25, 2.46)** | 1.37 (0.94, 1.99)  **1.67 (1.12, 2.50)** | 1.38 (0.98, 1.96)  **1.52 (1.05, 2.19)** | 1.15 (0.86, 1.54)  **1.64 (1.18, 2.27)** | 1.32 (1.00, 1.76)  **1.89 (1.39, 2.57)** | 1.21 (0.91, 1.60)  **1.49 (1.11, 2.00)** |
| Mother’s gambling child age 18  - no problem gambler  - low-high risk gambler | **1.55 (1.18, 2.03)**  1.07 (0.48, 2.38) |  |  | **1.61 (1.25, 2.07)**  **1.99 (1.02, 3.89)** | **1.45 (1.14, 1.84)**  1.88 (0.89, 3.98) | **1.36 (1.07, 1.75)**  1.32 (0.68, 2.57) |
| Financial difficulties pregnancy  - difficulty score >3 |  |  | **0.74 (0.54, 1.00)** |  |  |  |
